# Critical Issues in Mycobiota Analysis

**DOI:** 10.3389/fmicb.2017.00180

**Published:** 2017-02-14

**Authors:** Bettina Halwachs, Nandhitha Madhusudhan, Robert Krause, R. Henrik Nilsson, Christine Moissl-Eichinger, Christoph Högenauer, Gerhard G. Thallinger, Gregor Gorkiewicz

**Affiliations:** ^1^Institute of Pathology, Medical University of GrazGraz, Austria; ^2^Theodor Escherich Laboratory for Medical Microbiome Research, Medical University of GrazGraz, Austria; ^3^BioTechMed-Graz, Interuniversity CooperationGraz, Austria; ^4^Section of Infectious Diseases and Tropical Medicine, Department of Internal Medicine, Medical University of GrazGraz, Austria; ^5^Department of Biological and Environmental Sciences, University of GothenburgGothenburg, Sweden; ^6^Division of Gastroenterology and Hepatology, Department of Internal Medicine, Medical University of GrazGraz, Austria; ^7^Institute of Molecular Biotechnology, Graz University of TechnologyGraz, Austria

**Keywords:** microbiota, mycobiota, internal transcribed spacer (ITS), 16S rRNA gene, multiple sequence alignment (MSA), OTU picking, formalin-fixed paraffin-embedded tissue (FFPE), DNA isolation

## Abstract

Fungi constitute an important part of the human microbiota and they play a significant role for health and disease development. Advancements made in the culture-independent analysis of microbial communities have broadened our understanding of the mycobiota, however, microbiota analysis tools have been mainly developed for bacteria (e.g., targeting the 16S rRNA gene) and they often fall short if applied to fungal marker-gene based investigations (i.e., internal transcribed spacers, ITS). In the current paper we discuss all major steps of a fungal amplicon analysis starting with DNA extraction from specimens up to bioinformatics analyses of next-generation sequencing data. Specific points are discussed at each step and special emphasis is placed on the bioinformatics challenges emerging during operational taxonomic unit (OTU) picking, a critical step in mycobiota analysis. By using an *in silico* ITS1 mock community we demonstrate that standard analysis pipelines fall short if used with default settings showing erroneous fungal community representations. We highlight that switching OTU picking to a closed reference approach greatly enhances performance. Finally, recommendations are given on how to perform ITS based mycobiota analysis with the currently available measures.

## Introduction

It is now well-established that the microbiota contributes significantly to human health and disease. So far, microbiota investigations have been mainly focused on bacteria, but also archea, viruses, and micro-eukaryotes such as protozoa and fungi are part of human-associated microbial communities. Fungi are prevalent in all microbially colonized body habitats including skin, the gastrointestinal (GI)-, urogenital-, and respiratory tract (Charlson et al., 2012; Findley et al., 2013; Hallen-Adams et al., 2015). Up to now more than 390 fungal species have been described in humans (Oever and Netea, 2014; Gouba and Drancourt, 2015). Depending on the habitat the abundance of fungal cells varies from <0.1% of microorganisms in the GI tract to up to 10% on skin (Belkaid and Naik, 2013). An average fungal cell is about 100-fold larger than an average bacterial cell, which translates into a significant fungal biomass, providing abundant bioactive molecules to the host and shaping its physiology (Underhill and Iliev, 2014). The GI mycobiota actively interacts with the immune system, for instance through the human innate immune receptor Dectin-1 able to dampen GI inflammation (Iliev et al., 2012). A balanced mycobiota prevents from hyperinflammation of the GI tract and alterations in fungal community composition due to antifungal drugs exacerbate colitis in mice (Wheeler et al., 2016). In humans genetic defects in certain immune-regulatory genes (e.g., *STAT1, CARD9*, etc.) or Il-17 and Il-22 signaling pathways lead to severe fungal syndromatic infections, such as chronic mucocutaneous candidiasis or the APECED (Autoimmune Polyendocrinopathy, Candidiasis, Ectodermal Dystrophy) syndrome (Oh et al., 2013; Underhill and Iliev, 2014). Compositional mycobiota shifts are reported in various diseases (Cui et al., 2013) and also interdependencies between the fungal and bacterial component of the microbiota exist. They are exemplified by disease-specific inter-kingdom alterations, reported for instance in inflammatory bowel disease (IBD, Ott et al., 2008; Hoarau et al., 2016; Sokol et al., 2016) or in the lung microbiome of cystic fibrosis patients (Kim et al., 2015). Importantly, fungi contribute significantly to human infections, especially in immune-compromised, chronically ill and intensive care patients wherein the respiratory or GI tract are often the origins of fungal systemic infections (Brown et al., 2012; Krause et al., 2016).

## Internal transcribed spacers (ITS) as fungal molecular barcodes

Currently, amplicon-based next generation sequencing is the standard measure for the culture-independent assessment of the mycobiota. Also metagenomic approaches are increasingly used, providing functional insights into the mycobiota. However, their broad application is still too costly due to the required sequencing effort to capture the relatively rare fungal biosphere and the special needs for bioinformatics analysis paired with underdeveloped fungal reference genome databases make metagenomics approaches still cumbersome (Tang et al., 2015). Early culture-independent mycobiota investigations used the eukaryotic 18S ribosomal RNA gene, in analogy to the prokaryotic 16S rRNA gene, as molecular target enabling PCR amplification of fungal DNA and subsequent taxonomic profiling via sequence analysis (Simon et al., 1992; Kappe et al., 1996; Smit et al., 1999; Hunt et al., 2004). The 18S rRNA gene, however, is less discriminatory for fungi compared to its prokaryotic equivalent often failing to discriminate fungi at lower taxonomic levels, such as genus or species (Hartmann et al., 2010; Lindahl et al., 2013).

**Figure 1:**
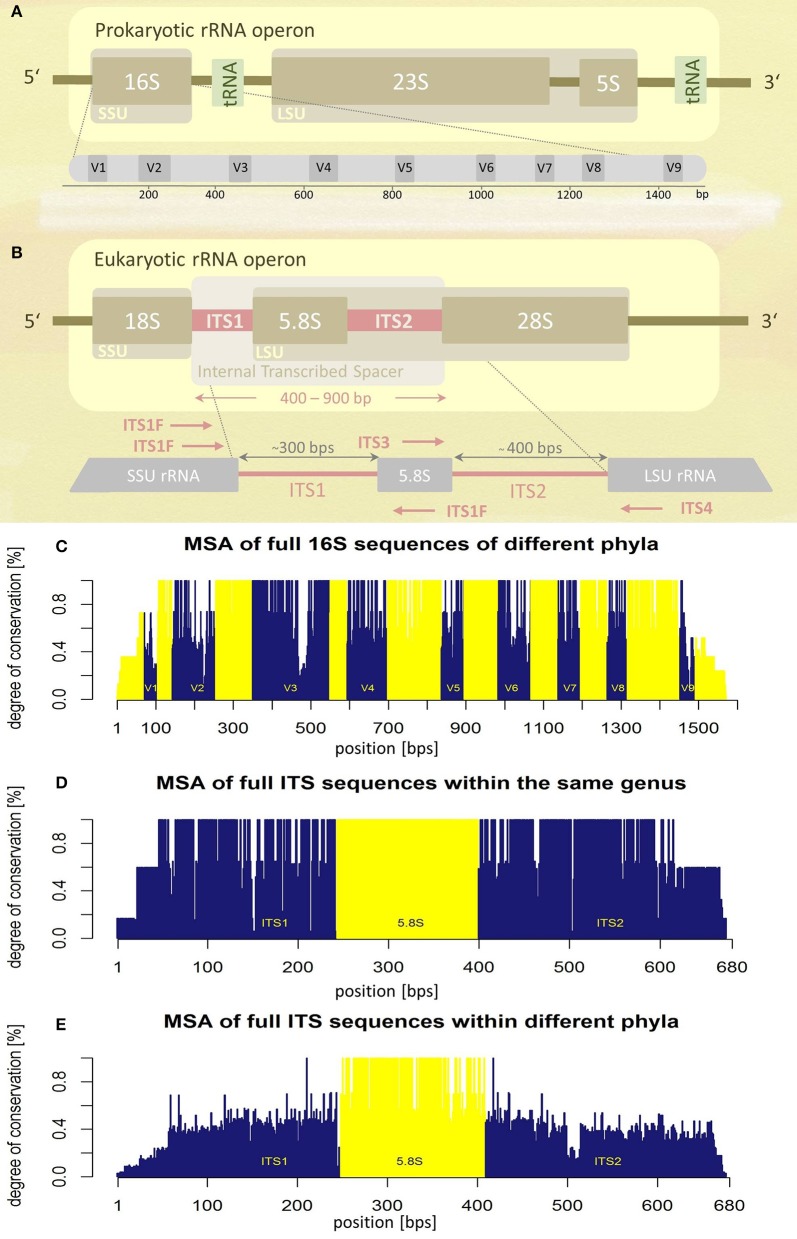
**Schematic representations of rRNA operons and their variability assessed by multiple sequence alignments (MSA). (A)** Prokaryotic and **(B)** eukaryotic rRNA operons. Position and orientation of oligonucleotide primers used for ITS amplification are schematically indicated (for sequence information see Table 1). SSU, small subunit; LSU, large subunit; tRNA, transfer RNA; V1-V9, variable regions; ITS, internal transcribed spacer; bps, base-pairs. **(C)** Multiple sequence alignment (MSA) of the entire 16S rRNA operon of five different bacterial species (encompassing five different phyla). Variable regions (V1–V9) are highlighted in blue, conserved regions in yellow, positions according to the *E. coli* 16S rRNA (GenBank acc. no.: J01695.2). **(D)** MSA of the complete internal transcribed spacer region of five different fungal species of the same genus (*Hydnum* sp.). **(E)** MSA of the complete ITS region of seven fungal taxa representing different phyla. Information about sequences used for MSA generation **(C,D)** is given as Supplementary Tables S3–S5.

The prokaryotic and the eukaryotic rRNA operons exhibit different genetic architectures (Figures 1A,B). The eukaryotic rRNA cistron consists of the 18S (small subunit, SSU), 5.8S, and 28S (large subunit, LSU) rRNA genes transcribed as a unit by RNA polymerase I, including two internal transcribed spacer regions, ITS1 and ITS2, flanking the 5.8S rRNA gene. The two ITS regions are post-transcriptionally removed and are absent in the mature ribosome. Since they are dispensable for ribosome function, they experience a lower evolutionary pressure leading to higher sequence variability (Figures 1C–E). The increased level of sequence variability enables discrimination of even closely related taxa (e.g., at species level). In addition ITS sequences seem to represent superior molecular targets for fungal PCR amplification compared to SSU and LSU sequences, signified by higher positive PCR amplification rates (Schoch et al., 2012). Based on these observations, the Fungal Barcoding Consortium recently denoted the ITS region as the universal barcode for fungi superior to other molecular markers (Schoch et al., 2012).

In the following sections, we discuss the main steps of amplicon-based mycobiota analyses with special emphasis on the bioinformatics challenges emerging if standard bioinformatics analysis pipelines such as mothur, QIIME, or MICCA are employed (Schloss et al., 2009; Caporaso et al., 2010; Albanese et al., 2015).

## Fungal DNA isolation

A variety of studies have shown that DNA isolation methods and oligonucleotide primer choice significantly influence the outcome of molecular phylogenetic surveys (Gorkiewicz et al., 2013; Tedersoo et al., 2014; Hallen-Adams et al., 2015). Numerous protocols and kits are available for isolation of fungal DNA and they follow similar basic principles with slight modifications dependent on the specimen type used (Paulino et al., 2006; Ghannoum et al., 2010; Findley et al., 2013; Lindahl et al., 2013; Gosiewski et al., 2014; Oh et al., 2014). The basic protocol involves mechanical cell disruption using bead beating, followed by enzymatic cell lysis. Especially the addition of lyticase, and endoglucanase hydrolyzing the covalent bounds between β-(1-3)-D-glucose molecules in the fungal cell-wall glycan, is an essential step to enable complete fungal cell lysis (Muñoz-Cadavid et al., 2010; Goldschmidt et al., 2014). The final DNA purification step is often performed by using membrane-based procedures (van Burik et al., 1998; Lindahl et al., 2013).

Aside of typically sampled native material (e.g., swabs, etc.) also other resources for mycobiota investigations exist. Formalin-fixed paraffin-embedded (FFPE) tissue samples play an important role in the clinical context. Biopsies or surgically removed tissues are typically fixed in formalin (10%) immediately after they are collected from the patient, thus they represent a well-preserved resource for the analysis of biomolecules including nucleic acids (Sangoi et al., 2009; Kocjan et al., 2015). FFPE specimens are typically used for diagnostic purposes (e.g., histopathology) but are also amenable for molecular scientific investigations. Their prevalence in biological repositories such as biobanks make them ideal specimens to study the mycobiota in the context of human disease (Yuille et al., 2008). About 70 commercially kits are available for DNA extraction out of FFPE material (Kocjan et al., 2015), however, nucleic acid isolation from FFPE material is challenging. Biomolecules are typically cross-linked and fragmented due to formalin, and factors such as the pH of the fixative, duration of fixation, and importantly the DNA extraction method applied greatly influence the quality of the extracted DNA (Bonin and Stanta, 2013; Kocjan et al., 2015). Factors such as residual formalin inhibiting proteinase K activity and omitting complete cell lysis, as well as the presence of PCR inhibitors in the DNA extract might altogether interfere with successful fungal DNA amplification (Coura et al., 2005; Muñoz-Cadavid et al., 2010).

**Figure 2:**
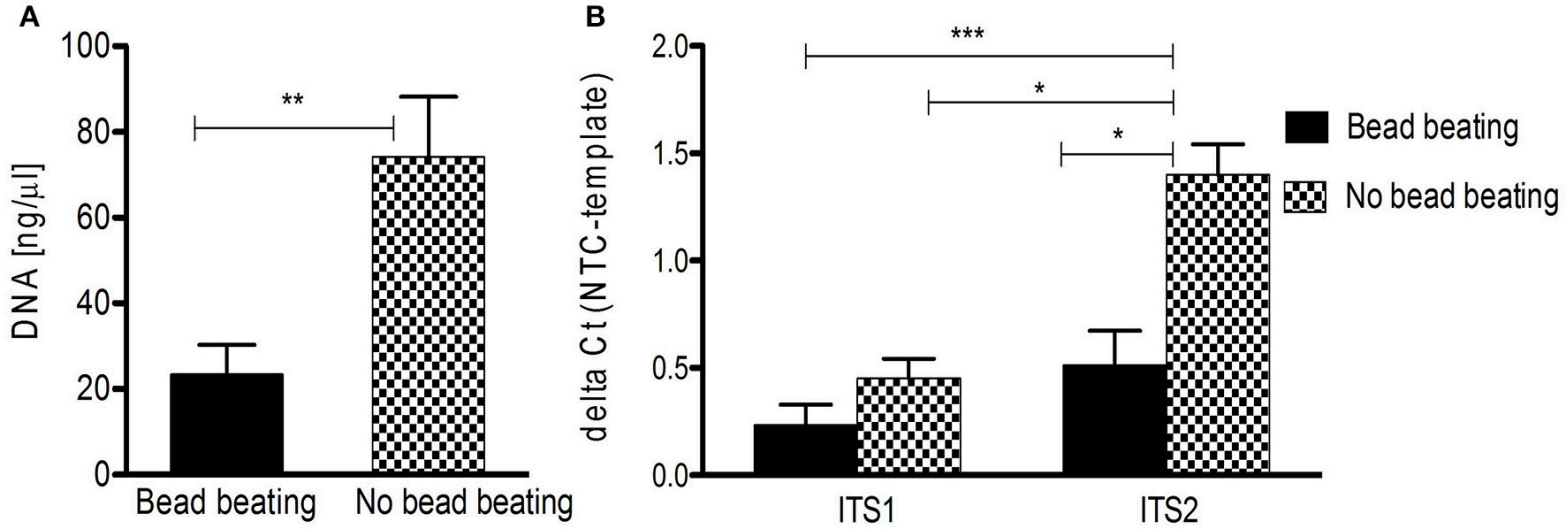
**DNA isolation from human FFPE skin samples and ITS PCR amplification influenced by beat beating. (A)** Significant difference in overall DNA yield from FFPE skin samples (*n* = 10) with and without bead beating (^**^p < 0.005 by Mann Whitney test; data are mean + SEM). **(B)** Significantly increased detection of fungal DNA isolated without bead beating by ITS2 qPCR (*n* = 10; ^*^p < 0.05, ^***^p < 0.005, Kruskal-Wallis test; data are mean + SEM). NTC, negative control.

These difficulties make a thorough review of the (pre-) analytical process of mycobiota studies mandatory. To highlight the influence of pre-analytics on ITS based mycobiota investigations we assessed the performance of DNA extraction from human skin FFPE samples (see Supplementary Table S1 for sample information) with a commercially available kit (QIAamp DNA FFPE tissue kit, Qiagen) reported to be efficient for fungal DNA extraction out of FFPE material (Muñoz-Cadavid et al., 2010). We added a mechanical cell disruption step (bead-beating) to the procedure (MagnaLyser, Roche), since this step was shown to be crucial for complete lysis of microbial cells in specimens, significantly influencing correct community representation (de Boer et al., 2010; Reck et al., 2015). A detailed description of the applied method is given in the Data Sheet S1. Interestingly, we observed that bead-beating significantly lead to lower DNA yields and a significantly decreased signal-to-noise ratio in ITS PCR, impairing efficient fungal PCR amplification (Figure 2). Thus, mechanical lysis of specimens could also counteract reliable mycobiota investigations especially if low-biomass samples such as skin are used.

## ITS amplification via PCR

For amplification of fungal DNA various primers have been designed targeting different regions of the rRNA operon or other marker genes encoding translation elongation factor 1-α, RNA polymerase II, β-tubulin, and the minichromosome maintenance complex component 7 (MCM7) protein (White et al., 1990; Tanabe et al., 2002; McLaughlin et al., 2009; O'Donnell et al., 2010; Schoch et al., 2012; Toju et al., 2012; Lindahl et al., 2013). Of these, the ITS regions are considered the formal barcode for fungal taxonomy (Schoch et al., 2012; Lindahl et al., 2013). As noted above, ITS1 and ITS2 sequences are highly variable and can be used to discriminate fungi even down to species level (Martin and Rygiewicz, 2005; Porras-Alfaro et al., 2014). However, each ITS primer combination fails to amplify certain species, a situation similar to bacterial 16S rRNA gene based analysis (Bellemain et al., 2010). Thus the use of multiple primer combinations and/or primers with degenerated nucleotide positions is recommended to capture the entire fungal community (Ihrmark et al., 2012; Toju et al., 2012). Table 1 summarizes commonly used ITS1 and ITS2 oligonucleotide primers. Of note, the ITS2 region was reported to perform better for fungal DNA amplification out of FFPE material (Muñoz-Cadavid et al., 2010; Flury et al., 2014). We also observed increased PCR performance using ITS2 primers and human skin FFPE samples (Figure 2B). However, other reports obtained similar amplification rates with ITS1 and ITS2 oligonucleotides (Mello et al., 2011; Bazzicalupo et al., 2013; Blaalid et al., 2013; Lindahl et al., 2013).

**Table 1:** **Overview of commonly used ITS1 and ITS2 oligonucleotide primer pairs**.

**Region**	**Name**	**Sequence (Forward)**	**Name**	**Sequence (Reverse)**	**Length (bp)**	**Tm (°C)**	**References**
ITS1	ITS1	TCCGTAGGTGAACCTGCGG	ITS2	GCTGCGTTCTTCATCGATGC	~290	65	White et al., 1990; Muñoz-Cadavid et al., 2010; Schoch et al., 2012
	ITS5	GGAAGTAAAAGTCGTAACAAGG	ITS2	GCTGCGTTCTTCATCGATGC	~315	63	White et al., 1990
	ITS1F	CTTGGTCATTTAGAGGAAGTAA	ITS2	GCTGCGTTCTTCATCGATGC	~350	51	Mello et al., 2011
	ITS1-F_KYO2	TAGAGGAAGTAAAAGTCGTAA	ITS2_KYO2	TTYRCTRCGTTCTTCATC	~300–400	47	Toju et al., 2012
	18S-F	GTAAAAGTCGTAACAAGGTTTC	5.8S-1R	GTTCAAAGAYTCGATGATTCAC	~300–400	^*^ns	Findley et al., 2013
ITS2	ITS3	GCATCGATGAAGAACGCAGC	ITS4	TCCTCCGCTTATTGATATGC	~330	62	White et al., 1990, Muñoz-Cadavid et al., 2010; Mello et al., 2011; Flury et al., 2014
	ITS3_KYO2	GATGAAGAACGYAGYRAA	ITS4	TCCTCCGCTTATTGATATGC	~400	47	Toju et al., 2012
	fITS9	GAACGCAGCRAAIIGYGA	ITS4	TCCTCCGCTTATTGATATGC	~390	55	Ihrmark et al., 2012
	fITS7	GTGAR TC ATC GAATC TTTG	ITS4	TCCTCCGCTTATTGATATGC	~340	57	Ihrmark et al., 2012
	gITS7	GTGARTCATCGARTCTTTG	ITS4	TCCTCCGCTTATTGATATGC	~340	56	Ihrmark et al., 2012
	5.8S-F	GTGAATCATCGARTCTTTGAAC	28S1-R	ATGCTTAAGTTCAGCGGGTA	~300	^*^ns	Findley et al., 2013
ITS1-2 incl. 5.8S rRNA gene	ITS5	GGAAGTAAAAGTCGTAACAAGG	ITS4	TCCTCCGCTTATTGATATGC	~641	58	White et al., 1990; Muñoz-Cadavid et al., 2010; Flury et al., 2014
	ITS1F	CTTGGTCATTTAGAGGAAGTAA	ITS4-B	CAGGAGACTTGTACACGGTCCAG	~600	55	Gardes and Bruns, 1993
	ITS1-F_KYO2	TAGAGGAAGTAAAAGTCGTAA	ITS4	TCCTCCGCTTATTGATATGC	~700	47	Toju et al., 2012

* *ns, not specified*.

## Bioinformatics challenges in mycobiota analyses

**Figure 3:**
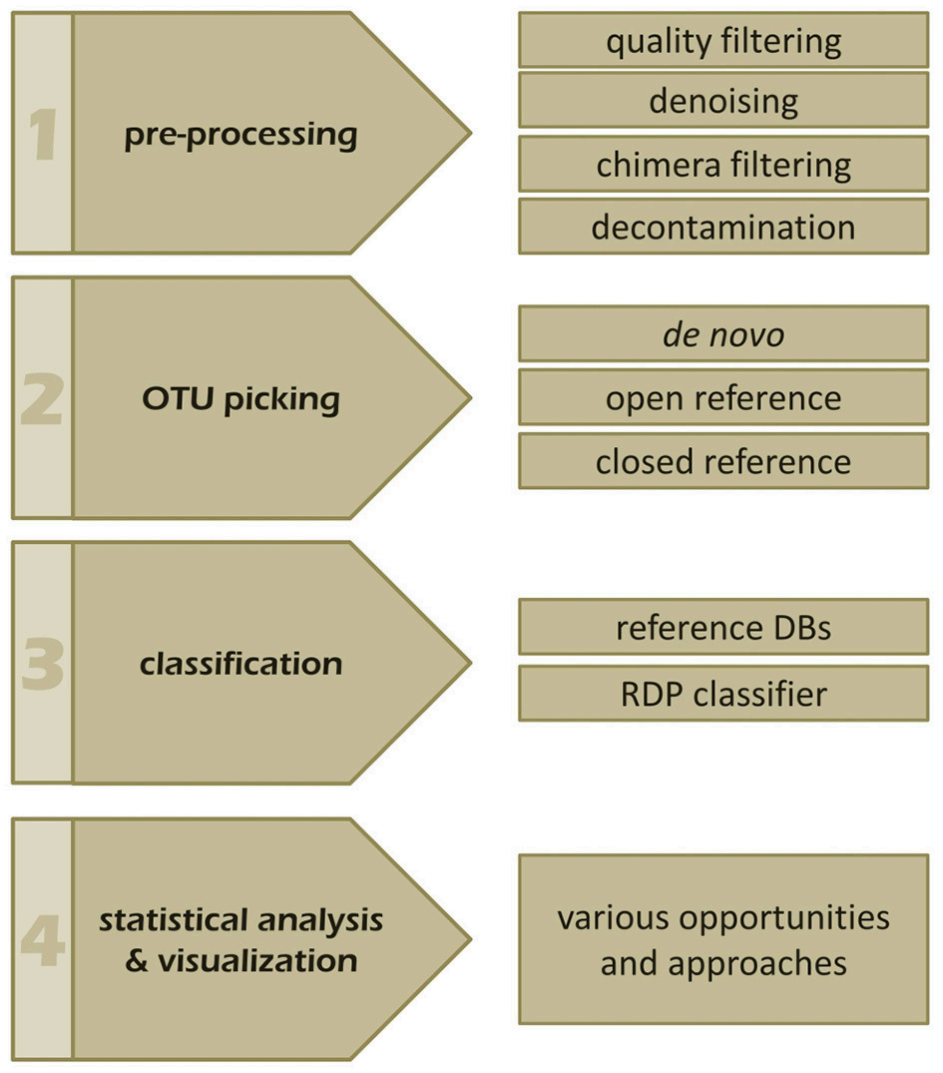
**The four main steps of a typical amplicon analysis workflow**. Individual steps and features of (1) pre-processing, (2) OTU picking, (3) taxonomic annotation, as well as, (4) visualization and statistics are indicated and discussed in the manuscript.

The bioinformatics analysis workflow of amplicon data can be summarized into four main steps: (i) pre-processing, (ii) OTU picking, (iii) taxonomic classification, and (iv) visualization and statistical analysis (Figure 3; Kuczynski et al., 2012). So far dedicated bioinformatics tools for mycobiota analyses are sparse. Measures originally developed for 16S rRNA gene data, like QIIME (Caporaso et al., 2010) and mothur (Schloss et al., 2009) are often employed to investigate ITS amplicons. However, these tools pose several shortcomings when applied to ITS sequences, especially when standard protocols are used. In the following the main analytical steps and potential hurdles of ITS based amplicon data analyses are discussed with special emphasis on OTU clustering (OTU picking) and classification. We also highlight the effect of different OTU picking strategies on taxonomic classification of ITS data by comparative analysis of an ITS1 *in silico* mock community.

## Pre-processing of amplicon raw data

Current pre-processing recommendations include rigorous length filtering of reads, noise reduction (detection, correction, and removal of sequencing errors and artifacts), quality filtering (removal of reads with quality scores below a defined threshold; average > 25), chimera removal (detection and removal of artificially created reads, produced different targets during PCR), as well as removal of singletons/doubletons (Bokulich et al., 2013). The latter could emerge due to sequencing errors (e.g., within homopolymers) leading to OTU inflation of data, which is dependent also on the sequencing technology used (Schirmer et al., 2015). Choice of pre-processing methods and used parameters heavily influence the number of created OTUs, which could lead to underestimation of species diversity if too stringent filtering is applied (Flynn et al., 2015; Kopylova et al., 2016). However, adequate pre-processing of raw reads is mandatory independent of the used maker gene, leading to a reduced number of assigned OTUs and less noise in the data. Basically we refer to the suggestions of Schloss et al. (2011), but as there are no general rules for pre-processing we strongly recommend looking carefully into what is happening during filtering rather than just applying default parameters.

## OTU picking—clustering into operational taxonomic units (OTUs)

**Figure 4:**
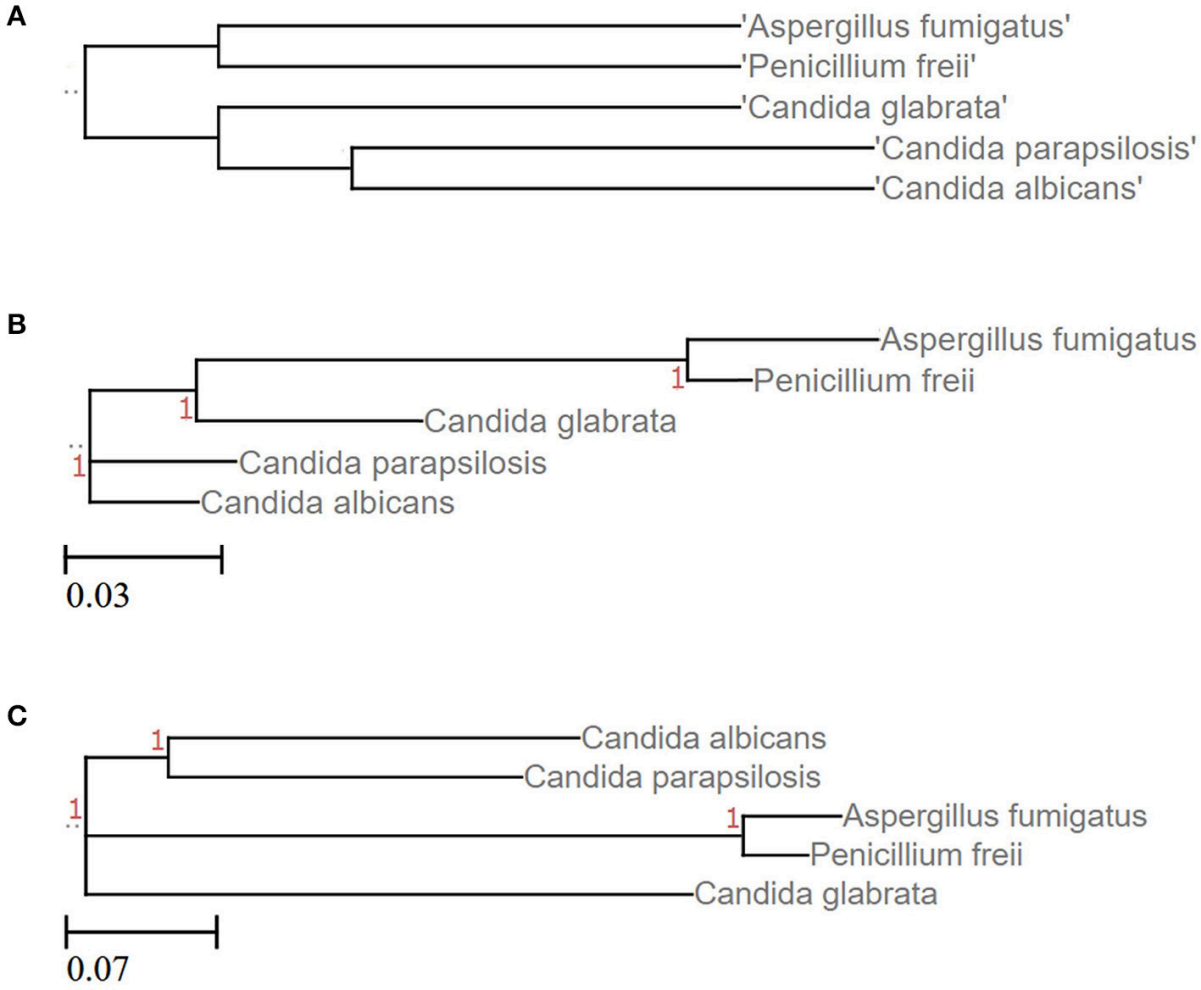
**Phylogenetic resolution of five different fungal species is impaired when clustering ITS sequences. (A)** Tree based on the corresponding NCBI taxonomy information using NCBI's Common Tree. Treeing is congruent with the phylogenetic study performed by Diezmann et al. (2004). **(B)** LSU based treeing recapitulates largely the NCBI taxonomy. **(C)** ITS based treeing impairs phylogeny. Trees of subfigures **(B,C)** are based on MSA of LSU and ITS2 fragments, respectively (taxon IDs and accession numbers are given as Data Sheets S3–S5).

Numerous approaches and tools are available for clustering sequences into OTUs. Current algorithms developed primarily for 16S rRNA gene amplicons are summarized in Table 2. In general OTU clustering and annotation could be achieved by using three different strategies (i) *de novo-*, (ii) closed reference-, and (iii) open reference-based clustering. Briefly, a closed reference approach calculates for each input sequence the best pairwise alignment to a pre-defined reference database collection. Sequences with the same best match are binned into the same cluster (i.e., OTU). In contrast, *de novo* based strategies cluster sequences within a pre-defined distance (commonly 3%). For each of these clusters a representative sequence is selected and taxonomically classified. Open-reference OTU picking is a mixture of both. Reads are first clustered using a closed reference approach and all reads which fail in this first step are subsequently clustered using a *de novo* strategy (Rideout et al., 2014; Westcott and Schloss, 2015). A recent comparison of the three different clustering strategies revealed the *de novo* approach based on a global distance matrix (implemented by default by mothur) as the optimal method for clustering 16S rRNA gene sequences into OTUs (Westcott and Schloss, 2015). Such benchmark comparisons are unfortunately missing for ITS amplicons. Importantly, the use of multiple sequence alignments (MSA) for clustering ITS sequences in a *de novo* approach poses a significant problem. ITS sequences show a high degree of intraspecific variation (Figures 1D,E), which leads to the introduction of gaps during the alignment process and subsequently to erroneous multiple sequence alignments exhibiting wrong phylogenetic resolution (Figure 4). In addition, there is no commonly accepted genus or species level cut-off for the formation of ITS clusters, such as 5% variation for genus- and 3% for species-level clustering applied to 16S rRNA gene data (Stackebrandt and Goebel, 1994). Often 3% variation is used and this cut-off seems to perform reasonable for fungal ITS sequences, although taxonomic resolution is clearly impaired within certain taxa. Both, ITS1 and ITS2, show a highly congruent fungal taxonomic resolution (Blaalid et al., 2013).

**Table 2:** **List of commonly used clustering algorithms**.

**Algorithm name**	**Algorithm type**	**Multiple sequence alignment required**	**Integrated in**	**References**
Mothur	Hierarchical	YES	Mothur	Schloss et al., 2009
UCLUST	Greedy	NO	QIIME	Edgar, 2010
UPARSE	Greedy	NO	QIIME	Caporaso et al., 2010; Edgar, 2013; Albanese et al., 2015
SWARM	Agglomerative	NO	QIIME, MICCA	Caporaso et al., 2010; Mahé et al., 2014; Albanese et al., 2015
OTUCLUST	Greedy	NO	MICCA	Albanese et al., 2015

## Taxonomic classification of OTUs

If a closed reference-based approach is used, taxonomic classification is achieved already during the OTU picking step, wherein OTUs represent clusters of identical matches to the reference database. If a *de novo* strategy is employed a proxy sequence from each cluster is chosen and taxonomically classified either by calculating sequence similarities between the proxy sequence and a reference database or by estimating the classification confidence using a pre-trained classifier, such as the RDP classifier (Wang et al., 2007). The latter one offers training sets for ITS (Porras-Alfaro et al., 2014) as well as for LSU (Liu et al., 2012) sequences. Accurate taxonomic classification of sequences requires reference databases of high quality. The UNITE (Unified system for the DNA based fungal species linked to the classification, https://unite.ut.ee) database for ITS fragments represents a curated full-length ITS sequence repository devoid of ambiguous sequences (Nilsson et al., 2014). Several factors lead to misannotated ITS sequences in repositories, such as GenBank, EMBL, or DDJB. For instance many fungi have sexual (teleomorph) and asexual (anamorph) forms and they are often classified as different taxa assigned even to different families (Mahé et al., 2012; Underhill and Iliev, 2014). UNITE represents currently the most comprehensive taxonomic ITS classification resource, providing ready-to-use application files for mothur, QIIME, and MICCA. Although still some fungal lineages are uncovered it comprises 536,881 sequence entries (as of January 2016, UNITE version 7.0). Recently, the hand curated ISHAM-ITS reference DNA barcoding database, with 3,200 sequences covering about 415 fungal species (as of December 2015) maintained by the Society for Human and Animal Mycology (ISHAM) was incorporated into UNITE (Irinyi et al., 2015). Noteworthy is UNITE's key concept, the so-called *species hypotheses* (SH). A SH represents an operational taxonomic unit at approximately species level (Kõljalg et al., 2013). Each SH is represented by the most homologous high quality sequence within a respective sequence cluster linked to a unique, permanent digital object identifier (DOI), which allows for unambiguous identification even in absence of a full formal taxonomic name or when a fungal OTU remains taxonomically unassigned. Of note, the global fungome is estimated to comprise 1.5–6 million different species (Hawksworth, 1991; Blackwell, 2011; Taylor et al., 2014), wherein currently 130,000 species are represented in the public sequence repositories (http://www.speciesfungorum.org/, accessed March 2016). These counts give already an idea about the “completeness” of the current fungal reference databases (Tedersoo et al., 2014).

## The effect of different OTU picking strategies on taxonomic classification of ITS data

**Figure 5:**
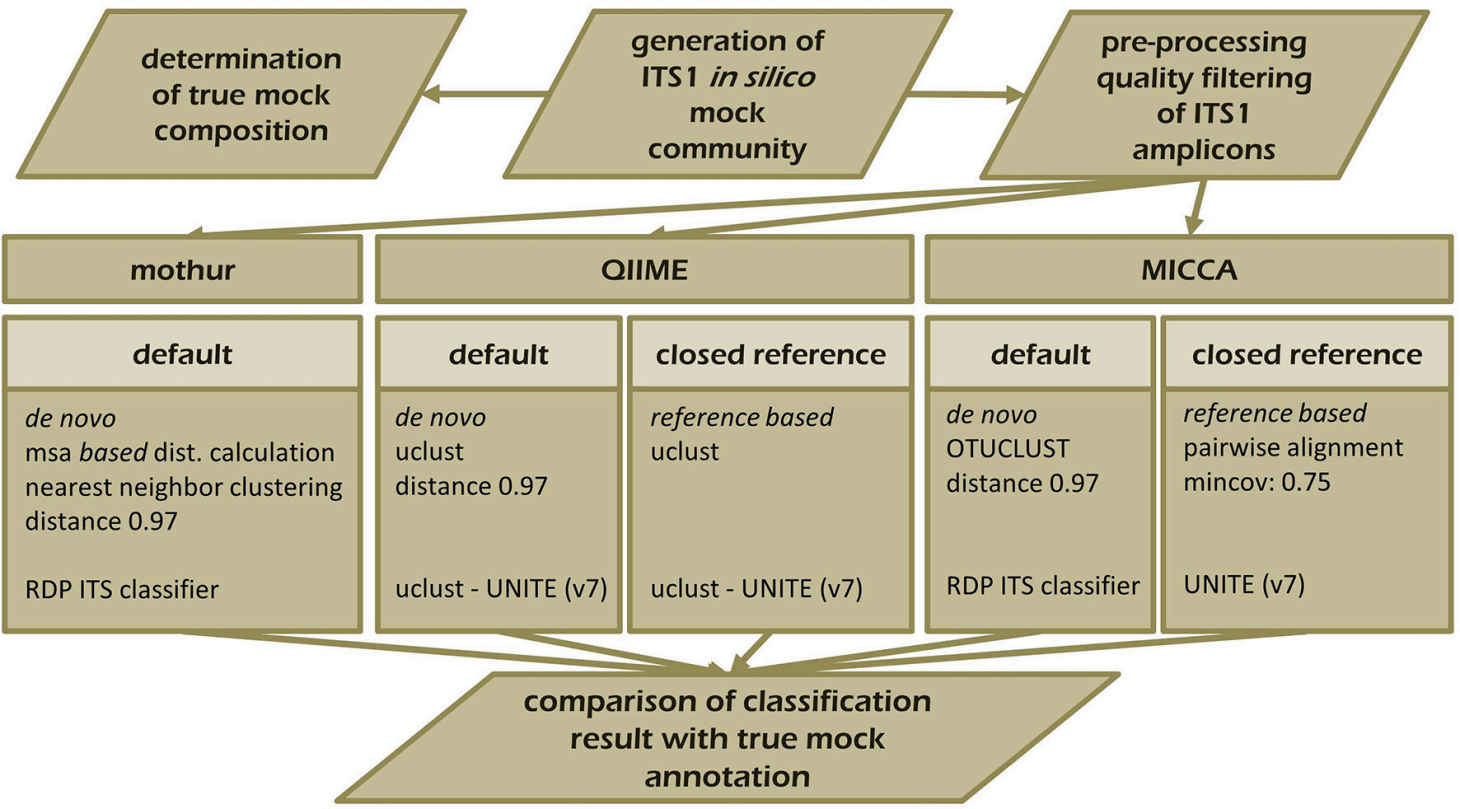
**Schematic overview of the experimental set-up testing the performance of mothur, QIIME, and MICCA to resolve the ITS1 mock community**. ITS1 fragments were extracted from the UNITE ITS reference collection (v.7) and analyzed with mothur (default workflow), QIIME, and MICCA (default and closed reference based workflow).

To demonstrate the influence of different OTU picking strategies on phylogenetic resolution of fungal communities we compared three commonly used analysis pipelines mothur, QIIME, and MICCA, employing an *in silico* created fungal ITS1 mock community. Therefore, 582,779 ITS1 fragments were extracted by ITSx (Bengtsson-Palme et al., 2013) from the public UNITE sequence collection (version 7, comprising 656,899 sequences). Amplicons were filtered for ambiguous lineage definitions, resulting in 345,201 sequences. These amplicons were quality filtered yielding finally 56,451 unique ITS1 fragments (accession numbers and taxonomic annotations are given in Supplementary Table S2). ITS1 fragments were subsequently clustered into OTUs by the default *de novo* strategies employed by mothur, QIIME, and MICCA, according to the standard protocol of each pipeline (for details see Data Sheet S1). For analyses with QIIME and MICCA, sequences were additionally binned into OTUs according to their taxonomic classification using a closed reference OTU picking strategy employing the UNITE database (version 7, 22.08.2016). The database was used for classification of representative sequences either directly for similarity-based comparisons or indirectly for training the RDP classifier. Finally, the assigned taxonomic classifications were compared to the true annotation of the ITS1 mock community. A scheme highlighting the experimental design and used parameters for comparison of pipelines is shown in Figure 5. Table 3 summarizes the comparison results, which clearly indicates that choice of the OTU picking strategy severely impacts the phylogenetic resolution of the ITS mock community. All pipelines used with default parameters failed to accurately classify the mock community down to species level. All approaches classified ITS1 reads with a reasonable accuracy only to the order level (range 87.61–97.34% correct assignment), except QIIME with default settings (*de novo*), which behaved poor (classifying only 33.53% of sequences correctly at phylum level and 0.07% at species level). A high number of singletons emerged by using all three *de novo* approaches, leading to OTU inflation, and wrongly clustered OTUs. Importantly, changing the default OTU picking approach of QIIME (*de novo*) to a closed reference approach increased the amount of correctly classified species to 71.62% (Table 3). Taken together these data indicate that closed reference based strategies should be preferred if ITS amplicons are analyzed. Nevertheless, a relatively large fraction of wrongly annotated OTUs might still persist, thus manual correction of taxonomic assignments (i.e., by individual blast analysis of sequences) might still improve classification (Iliev et al., 2012).

**Table 3:** **Correct classification of the ***in silico*** ITS1 mock community with different analysis pipelines and OTU picking strategies (% in parenthesis)**.

	**No. of classified sequences**	**No. of OTUs**	**Taxonomic classification**					
			**Phylum**	**Class**	**Order**	**Family**	**Genus**	**Species**
*In silico* ITS1 mock	56,451	11,336	6	45	143	409	1,931	11,336
Mothur	36,255	26,965	35,675 (98.40)	33,686 (92.91)	32,949 (90.88)	29,739 (82.02)	24,000 (66.20)	11,233 (30.98)
QIIME default	56,451	19,779	18,930 (33.53)	14,316 (25.36)	4,865 (8.62)	669 (1.18)	372 (0.65)	37 (0.07)
QIIME closed reference	31,676	8,764	31,504 (99.47)	31,284 (98.77)	30,832 (97.34)	29,211 (92.23)	26,650 (84.14)	19,246 (71.62)
MICCA default	56,446	20,878	54,698 (96.90)	51,095 (90.52)	49,454 (87.61)	44,511 (78.56)	36,549 (64.75)	19,246 (29.73)
MICCA closed reference	52,475	9,942	52,129 (99.34)	49,670 (94.65)	49,434 (94.20)	45,784 (87.25)	41,206 (78.52)	26,400 (50.31)

## Visualization and statistical analysis of ITS data

Visualization and statistical analyses of mycobiota data typically enable measures for community structure, such as alpha-diversity metrics (e.g., richness, evenness, Shannon index), as well as taxonomic turnover (i.e., changes in microbial composition between conditions or groups) called beta-diversity, which can be calculated with different distance measurements (Bray Curtis, Andernberg, UniFrac, etc.). Principle coordinates analysis (PCoA) plots based on these distance matrices enable simplified visualization of the structural resemblance of mycobiota profiles. Statistical identification of differential abundant taxa between groups could be achieved using tools such as LEfSe (Segata et al., 2011) or linear modeling approaches, such as DESeq (Paulino et al., 2006) or edgeR (Robinson et al., 2010). Measures for alpha- and beta-diversity are readily provided by tools such as mothur and QIIME and operate on the created OTU tables. Caution must be taken if measures derive phylogenetic information based on diversity matrices emerging from MSAs of ITS reads, such as UniFrac (Lozupone et al., 2011). Such methods lead to erroneous results because of the bad performance of aligning ITS reads as shown above (Figure 4).

## Conclusion

**Figure 6:**
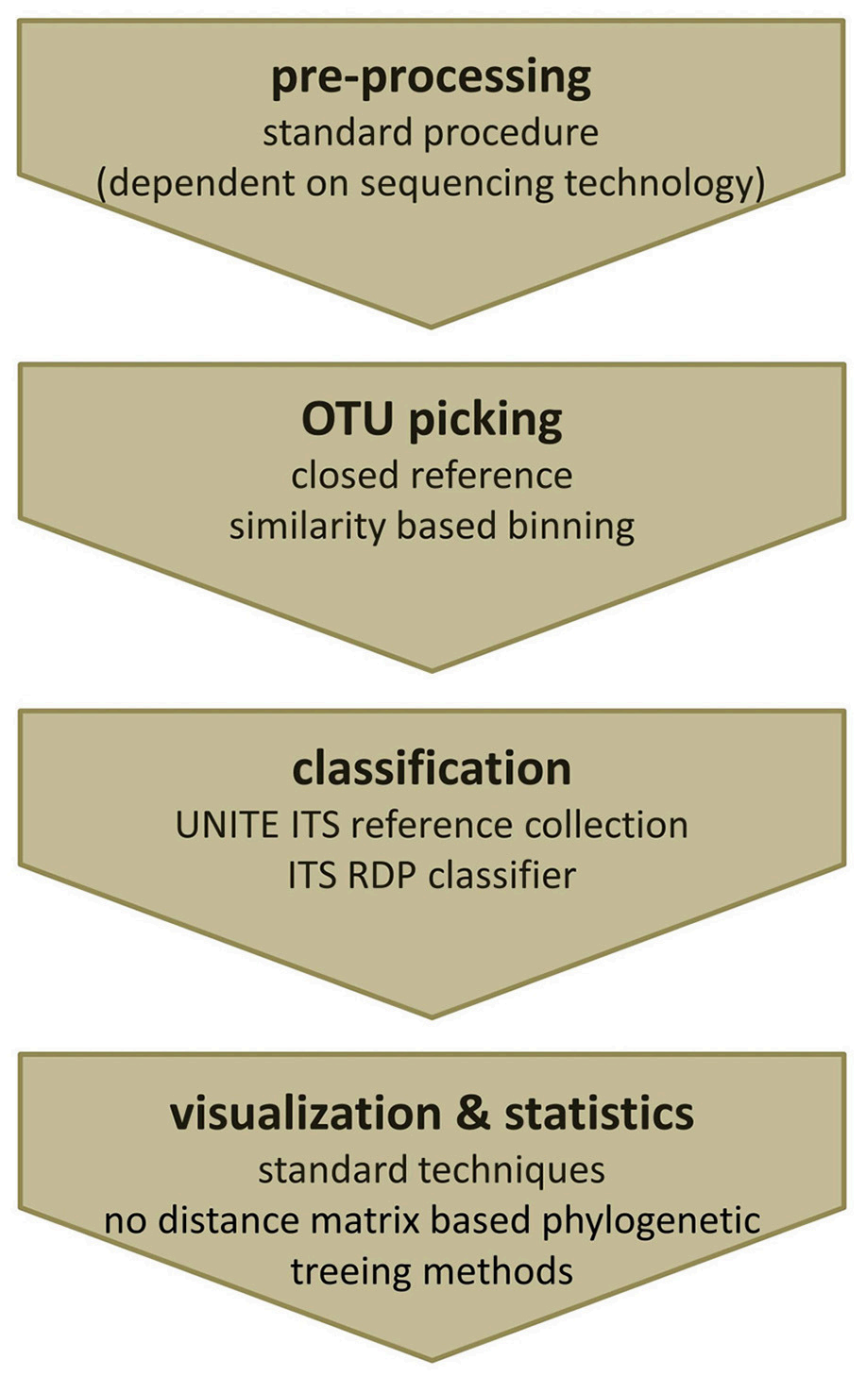
**Recommended workflow to analyze ITS amplicons**. (i) Pre-processing of fungal ITS amplicons can be performed using standard tools. (ii) For OTU picking a closed reference strategy is needed. (iii) Classification can either be done using the clustering information from the used reference database or by re-classification of representative reads using the ITS RDP classifier. (iv) Obtained OTU profiles (OTU tables) can be further analyzed by common visualization and statistical analysis techniques, except phylogenetic treeing methods based on distance matrices.

Fungal amplicon studies benefit greatly from the advancements made in the analysis of bacterial communities, nonetheless, many hurdles need still to be solved and standards are waiting to be defined. Although numerous protocols and kits are available for fungal DNA isolation out of complex specimens such as human tissue, protocols need to be adapted to the special study needs. Recommendations on how to perform ITS analyses using mothur and QIIME with non-phylogenetic diversity metrics have been recently released (e.g., https://mothur.org/wiki/Analysis_examples#Sanger_16S-ITS_rRNA_sequence_analysis, accessed February 2017, http://qiime.org/1.7.0/tutorials/fungal_its_analysis.html, accessed April 2016). Based on our experience, pre-processing, and quality filtering of ITS sequencing data, as well as chimera filtering could be done with standard 16S rRNA gene based procedures. We use the default workflow of mothur for ITS data pre-processing, assembling of paired reads, length-, quality-, and chimera filtering, as well as noise reduction as described in the MiSeq 16S SOP of Kuczynski et al. (2012, accessed May 2016). Since mothur employs pair-wise distance matrices, which require the creation of multiple sequence alignments, we recommend switching to tools such as QIIME or MIICA for further analyses, which allow for closed reference-based approaches. Subsequently QIIME can be used for visualization of mycobiota data. The crucial step within QIIME is to suppress tree generation within the OTU picking step and to use closed reference OTU picking instead of the default *de novo* strategy. The pre-formatted version of the UNITE ITS reference database which is provided directly by UNITE works perfectly with one of the reference-based OTU picking scripts of QIIME and MICCA. Alternatively sequences can be also classified and binned based on the information gained by the RDP classifier trained for ITS fragments or simply by an individual blast approach. A final summary of the recommended analysis steps for ITS based mycobiota analysis is given in Figure 6.

## Author contributions

Conceptualization: BH, RN, GT, and GG. Data analysis: BH and NM. Manuscript draft: BH and GG. Final manuscript and approval: All authors.

## Funding

This work was supported by BioTechMed-Graz and the Austrian Science Fund (FWF W1241-B18).

### Conflict of interest statement

The authors declare that the research was conducted in the absence of any commercial or financial relationships that could be construed as a potential conflict of interest.
