# Supplementary material for: Critical Issues in Mycobiota Analysis
Source: Front Microbiol. 2017 Feb 14;8:180. doi: 10.3389/fmicb.2017.00180 (PMC5306204; doi:10.3389/fmicb.2017.00180)
Supplement: Supplementary file 6 [file DataSheet1.pdf]

Supplementary Material

Critical issues in mycobiota analysis

Bettina Halwachs[1, 6, 7*], Nandhitha Madhusudhan[1, 6], Robert Krause[2], R. Henrik Nilsson[3], Christine Moissl-Eichinger[2, 7], Christoph Högenauer[4, 6, 7], Gerhard G. Thallinger[5, 7], Gregor Gorkiewicz[1, 6, 7*]

[1] Institute of Pathology, Medical University of Graz, Auenbruggerplatz 25, 8036 Graz, Austria

[2] Section of Infectious Diseases and Tropical Medicine, Department of Internal Medicine, Medical University of Graz, Auenbruggerplatz 15, 8036 Graz, Austria

[3] Department of Biological and Environmental Sciences, University of Gothenburg, Box 461, 405 30 Gothenburg, Sweden

[4] Division of Gastroenterology and Hepatology, Department of Internal Medicine, Medical University of Graz, Auenbruggerplatz 15, 8036 Graz, Austria

[5] Institute of Molecular Biotechnology, Graz University of Technology, Petersgasse 14, Graz, Austria

[6] Theodor Escherich Laboratory for Medical Microbiome Research, Medical University of Graz, Austria

[7] BioTechMed, Interuniversity Cooperation, Graz, Austria

*** Correspondence:** Corresponding Authors: bettina.halwachs@medunigraz.at; gregor.gorkiewicz@medunigraz.at; Institute of Pathology, Medical University of Graz, Graz, Auenbruggerplatz 25, 8036 Graz, Austria. Phone: +43-316-385-83649; Fax: +43-316-385-13432.

1 Supplementary Materials and Methods

The following subsections describe the protocols and workflows mentioned in the main text in detail, including used commands, parameters, and settings.

1.1 DNA isolation protocol for formalin fixed paraffin embedded samples

Formalin fixed paraffin embedded (FFPE) skin samples originated from a different study performed at the Medical University of Graz and DKFZ Essen, Germany (Prof. DDr. Jürgen Becker; institutional review board approval 24-167 ex 11/12). Values represented in the current paper are derived from the optimization of DNA extraction and ITS PCR. Sample information is given in supplementary Tab. S1.

DNA was extracted using the QIAamp DNA FFPE Tissue kit (Qiagen, Chatsworth, CA, USA) with modifications from the protocol reported by Munoz-Cadavid et al. (2010). The modifications were as follows:

a. 20 sections of FFPE material (each 5 µm) used.
b. Deparaffinization was performed with xylene with centrifugation for 10 mins at 13,000 rpm.
c. Use Magna Lyser tubes (Roche Diagnostics, Mannheim, Germany) with and without beads (green tubes; 1.4 mm ceramic beads). Two times homogenization with MagNA Lyser Instrument (Roche) at 6,000 rpm for 30 sec, cool sample in cooling block (-20°C) after each lysis.
d. The tissue pellet was digested with ATL buffer (Qiagen, Chatsworth, CA, USA) and treated with proteinase K solution at 56°C overnight. Subsequently, samples were processed using columns as per the manufacture's protocol.

1.2 Quantitative Real-Time PCR:

DNA extracted from FFPE skin samples was used to detect the presence of fungi. The ITS1 region was amplified using the primers ITS1-F CTTGGTCATTTAGAGGAAGTAA and ITS2-R GCTGCGTTCTTCATCGATGC; ITS2 region was amplified using ITS3-F GCATCGATGAAGAACGCAGC and ITS4-R TCCTCCGCTTATTGATATGC. Quantitative real-time PCR was performed with the CFX96 detection system (Bio-rad, Munich, Germany) using LuminoCt® SYBR® Green qPCR ReadyMix™ (Sigma-Aldrich, Steinheim, Germany). 50 ng of template DNA was added to each reaction with a final volume of 20 µl. The

amplification program included incubation of the reaction mixture for 3 mins at 95°C, followed by 40 cycles of 5 sec at 95°C, 15 sec at 51°C and finally a melt curve analysis by gradually increasing the temperature by 0.5°C from 65°C to 95°C with simultaneous recording of fluorescence signals. For detection of fungal DNA in skin samples, 50 ng of total DNA extracted from the FFPE material was used as a normalized input PCR. The CT values obtained for the samples were subtracted from the no template control (△ CT). Each measurement was performed in triplicates.

1.3 Statistical analysis of qPCR data

Quantitative PCR data were assessed with Shapiro-Wilk normality test for their normal distribution. Data are given as mean ± standard error of the mean. Statistical analyses were performed with GraphPad Prism 5 software, by the use of one-way ANOVA and Dunnett's post hoc test for multiple comparisons. *P*-values <0.05 were considered statistically significant.

1.4 *In silico* ITS1 mock community generation

The *in silico* ITS1 mock community is based on the publicly available UNITE ITS collection (version 7, 22.8.2016; Abarenkov et al. 2010) comprising 656,899 sequences. ITS sequences, consisting of the entire ITS1, 5.8S, ITS2 regions and partial 18S and 28S regions, were used as input for ITSx (version 1.0.11; Bengtsson-Palme et al. 2013) to computationally detect and extract the ITS1 region; 582,779 ITS1 sequences were derived. Undefined taxa containing *"unidentified"*, *"Incertae_sedis"*, or *"s__Fungi_*sp.*"*, were identified by a custom R script and subsequently removed prior to pre-processing (`remove.seqs` by mothur). The remaining 345,204 ITS1 fragments were quality filtered by mothur (version 1.36.1, Schloss et al. 2009) using `screen.seqs`. Specifically, sequences below a min length of 200, exceeding max length of 400, containing ambiguous or not determined bases (N), or more than 8 homopolymers were excluded from the sequence set. The obtained 93,661 sequences were subsequently uniqued by mothur's `unique.seqs`, resulting to 56,451 unique quality filtered ITS1 fragments. The final mock community comprises 6 phyla, 45 class, 143 order, 409 family, 1,931 genus, and 11,336 species, Tab. 3. This ITS1 collection is referred to as *in silico* ITS1 mock community (`its1Mock.fasta`) throughout the whole manuscript. Accession numbers and full linage information of these sequences are available in supplementary Tab. S2. Used commands of ITSx and mothur are listed below:

```
//extract ITS1 and ITS2 by ITSx from UNITE (version7)
```

```
ITSx -i UNITE_public_22.08.2016.fasta -o its1Mock --save_regions 'ITS1, ITS2' --cpu 20

//quality filtering, pre-processing using mothur
screen.seqs(fasta=its1Mock.fasta, maxambig=0, maxlength=400, minlength=200, maxhomop=8,
maxn=0)

//unique sequence set using mothur
unique.seqs(fasta=its1Mock.good.fasta)
```

1.5 Bioinformatical analysis of ITS1 fragments

To demonstrate the differences between *de novo* OTU picking strategies and closed reference based approaches the same set of ITS1 fragments was analyzed with mothur (Schloss et al. 2009), QIIME (Caporaso et al. 2010), and MICCA (Albanese et al. 2015) in default, *de novo*, as well as in closed reference mode. Details and commands for each tool are given in detail in the following sections. Unless otherwise specified, standard values and settings have been used with the applied commands.

1.5.1 mothur – default *de novo* OTU picking

In general the analysis followed the MiSeq SOP of Kozich et al. (2013, accessed May 2016) starting with `align.seqs`, since amplicons were already pre-processed (described within section 1.4). In the absence of an available reference sequence alignment, an alignment was manually created based on UNITE (version 6, 2014-12-30). Briefly, pre-clustered and pre-formatted version of UNITE for mothur was applied to ITSx to extract ITS1 fragments only. The Given taxonomic classification was used to select only one representative per species. According to this criteria a subset of 5,699 ITS1 fragments remained and were finally aligned by muscle version 3.8.31 (Edgar 2004) for further usage with mothur (*Note: we do not recommend to create your own MSA reference database for ITS fragments. This was just performed to proceed with the default analysis method of mothur to demonstrate its flaws based on MSA.*). Steps such as chimera detection and removal and deletion of contaminating sequences were skipped because of the *in silico* dataset. All used commands and parameters are given below:

```
//align mock sequences to reference alignment
```

```
align.seqs(fasta=its1Mock.fasta, reference=myITSRefAlignment.fasta)

//calculate pairwise distances
dist.seqs(fasta=its1Mock.align, cutoff=0.20)

//cluster sequences according to distance information
cluster(column=its1Mock.dist, name=its1Mock.names)

//use the cluster output to get representative sequences for classification with the RDP
classifier
get.oturep(column=its1Mock.dist, name=its1Mock.names, fasta=its1Mock.fasta,
list=its1Mock.fn.list, label=unique-0.03-0.05-0.10)

//assign taxonomic classification using the RDP classifier version 2.12 for fungal ITS
sequences
java -Xmx150g -jar /home/qiime/qiime_software/rdp_classifier_2.12-
release/dist/classifier.jar -o mothurMock_mothur_rdp_tax.txt -g fungalits_unite
its1Mock.0.03.rep.fasta
```

1.5.2 QIIME – default *de novo* OTU picking

The *in silico* ITS1 mock community was clustered into OTUs using QIIME's (version 1.8.0) `pick_otus.py` (http://qiime.org/1.8.0/scripts/pick_otus) script with default settings (clustering methods: uclust, cluster distance: 0.03, complete list of default settings is available via the given link). Subsequently, for each created OTU a representative sequence (proxy) was selected from the raw input sequence set, by `pick_rep_set.py` (http://qiime.org/1.8.0/scripts/pick_rep_set). Finally the selected representative sequences were classified using the `assign_taxonomy.py` (http://qiime.org/1.8.0/scripts/assign_taxonomy) script (method: uclust) using the pre-formatted and -clustered (97% identity) QIIME version of UNITE version 7, 22.08.2016.

```
//perform default de novo OTU picking using uclust with QIIME
pick_otus.py -i its1Mock.fasta -o qiime_denovo_default

//pick proxy for each newly created OTU
pick_rep_set.py -i its1Mock_otus.txt -f its1Mock.fasta

//assign taxonomic classification to each proxy sequence using the
//QIIME formatted, pre-clustered (97%) version of UNITE version7, 22.08.2016.
assign_taxonomy.py -i its1Mock.fasta_rep_set.fasta -t
sh_taxonomy_qiime_ver7_97_22.08.2016.txt -r sh_refs_qiime_ver7_97_22.08.2016.fasta
```

1.5.3 MICCA – default *de novo* OTU picking

MICCA was applied on the pre-processed ITS1 mock community sequences according to the specifications described in the documentation (http://micca.org/docs/latest/commands/otu.html, accessed September 2016) for *de novo* OTU picking. Sequences were clustered into OTUs and subsequently classified (http://micca.org/docs/latest/commands/classify.html, accessed September 2016) with the RDP classifier. Used commands for both steps are given below:

```
//de novo OTU picking
//cluster distance 0.03 (-d 0.97)
//minimum OTU size 1 read (-s 1)
micca otu -i its1Mock.fasta -o denovo_greedy_otus -d 0.97 -s 1

//classify taxonomies using the ITS RDP classifier
micca classify -m rdp --rdp-gene fungalits_unite -i denovo_greedy_otus/otus.fasta -o
denovo_greedy_otus/taxa.txt
```

1.5.4 QIIME – closed reference OTU picking

The *in silico* ITS1 mock sequences were analyzed using QIIME (version 1.8.0) using modified settings for closed reference OTU picking. Exact commands are given in the box below. Reads were clustered into OTUs using `pick_otus.py` with blast as method.

```
//perform closed reference OTU picking by QIIME
//method: blast
//reference db: sh_refs_qiime_ver7_97_22.08.2016.fasta
//similarity threshold 80%
//minimal OTU size: 1 read
pick_otus.py -i its1Mock.fasta -o qiime_closed_ref -m blast -r
sh_refs_qiime_ver7_97_22.08.2016.fasta -s 0.80 -g 1

//pick proxy for each binned OTU
pick_rep_set.py -i its1Mock_otus.txt -f its1Mock.fasta
```

For each OTU created by the closed reference approach, taxonomic classification was added according to the given identifier and the corresponding taxonomy file `sh_tax_qiime_ver7_97_22.08.2016.txt` by a custom R script for further comparison with the true annotation.

1.5.5 MICCA – closed reference OTU picking

Pre-processed *in silico* ITS1 amplicons were used for closed reference OTU picking with MICCA as recommended within the tool documentation (http://micca.org/docs/latest/commands/otu.html, accessed September 2016). QIIME formatted and pre-clustered (97% identity) UNITE, version 7 (`sh_refs_qiime_ver7_97_22.08.2016.fasta`) was used as closed reference database. Taxonomic classification was assigned according to the given taxonomic reference database. Used commands and settings are listed below:

```
//closed reference OTU picking
micca otu -i its1Mock.fasta --method closed_ref --threads 20 -n 0.80 --ref -o
closed_ref_otus sh_refs_qiime_ver7_97_22.08.2016.fasta
```

1.6 Evaluation of the classification results

For all obtained classification results, three methods, two strategies, resulting in five different analyses and classifications, a comparison with the true linage information at each level was performed using a custom R script and a string compare function. The result of this comparison is discussed in the main manuscript and summarized in Tab. 3.

1.7 Generation of ITS and 16S multiple sequence alignment (MSA; see Fig. 1)

Complete ITS sequences from different fungal species were extracted manually from GenBank (Sayers et al. 2009). Taxonomic information, linage description and NCBI accession numbers are given in supplementary Tab. S4 and S5. Full 16S sequences were manually extracted from the Greengenes database release May, 2013 (DeSantis et al. 2006) and summarized in supplementary Tab. S3. The 16S rRNA gene sequence of Escherichia coli (GenBank, accession no: J01695.2) was added as reference for annotation of hypervariable regions. Multiple sequence alignments were created using MAFFT version 7.215 (Katoh and Standley 2013) via the EMBL-EBI analysis tool framework (Goujon et al. 2010), and visualized using EMBL-EBI MView (Brown, Leroy, and Sander 1998), supplementary data sheet S2A-C. For different bacterial phyla the created MSA visualize nicely the alterations of conserved and hypervariable regions throughout the whole 16S gene, supplementary data sheet S2A. In contrast, for different phylum level fungi only conservation around the 5.8S is detectable, which makes the MSA of distinct fungal ITS fragments meaningless, supplementary data sheet S2C. For ITS fragments of the same genus the ratio between conservation and variation allows even for species discrimination supplementary data sheet S2B.

1.8 Phylogenetic tree generation (Fig. 4)

Phylogenetic trees (Cladogram representation) were generated by either NCBI‘s Taxonomy Common Tree function (Fig. 4A, Sayers et al. 2009) based on the respective sequences or by the ClustalX version 2.1 phylogeny tree functionality (Larkin et al. 2007) based on the respective MSA, Fig. 4B-C. The phylogenetic tree information was exported to newick format (Olsen 1990) and visualized by the ETE Toolkit (http://etetoolkit.org/treeview/), Phylogenetic tree (newick) viewer (Huerta-Cepas, Serra, and Bork 2016).

1.9 Generation of multiple sequence alignment histograms (Fig. 1C-1E)

16S and ITS amplicons were aligned using ClustalX 2.1 (Larkin et al. 2007) to obtain q-score information for each position of the alignment. Q-scores have been imported into R (version 3.2.4) for visualization using the `barplot` function of the `graphics` package (R Development Core Team 2008).

2 Supplementary Tables and Files

Supplementary Table S1: Sample identification and DNA extraction yield with and without beat beating.

supplementary_table_S1.xlsx

Supplementary Table S2: Accession numbers and full annotation for all sequences of the *in silico* mock community.

supplementary_table_S2.xlsx

Supplementary Table S3: Summary and detailed information of full 16S sequences of different phylum level bacteria.

supplementary_table_S3.xlsx

Supplementary Table S4: Summary and detailed information of full ITS sequences of *Hydnum* genus.

supplementary_table_S4.xlsx

Supplementary Table S5: Summary and detailed information of full ITS sequences of different phylum level fungi.

supplementary_table_S5.xlsx

Data Sheet. S1: Supplementary Material and Methods: Critical issues in mycobiota analysis

supplementary_data_sheet_S1.pdf

Data Sheet. S2: Detailed multiple sequence alignments (html representation) generated by MAFFT of Fig. 1C-E.

supplementary_data_sheet_S2.zip

Data Sheet. S3: Fasta file comprising accession numbers and 28S sequences used for phylogenetic Tree generation of Fig. 4B.

supplementary_data_sheet_S3.fasta

Data Sheet. S4: Fasta file comprising accession numbers and ITS2 sequences used for phylogenetic Tree generation of Fig. 4C.

supplementary_data_sheet_S4.fasta

Data Sheet. S5: NCBI Taxonomy entries used within the NCBI Common Tree, Fig. 4A .
supplementary_data_sheet_S5.zip

3 References

Abarenkov, K. et al. (2010). The UNITE database for molecular identification of fungi - recent updates and future perspectives. *New Phytol.* 186, 281-285.

Albanese, D., Fontana, P., De, F.C., Cavalieri, D., and Donati, C. (2015). MICCA: a complete and accurate software for taxonomic profiling of metagenomic data. *Sci. Rep.* 5, 9743.

Bengtsson-Palme, J. et al. (2013). Improved software detection and extraction of ITS1 and ITS2 from ribosomal ITS sequences of fungi and other eukaryotes for analysis of environmental sequencing data. *Methods Ecol. Evol.* 4, 914-919.

Brown, N.P., Leroy, C., and Sander, C. (1998). MView: a web-compatible database search or multiple alignment viewer. *Bioinformatics* 14, 380-381.

Caporaso, J.G. et al. (2010). QIIME allows analysis of high-throughput community sequencing data. *Nat. Methods* 7, 335-336.

DeSantis, T.Z. et al. (2006). Greengenes, a chimera-checked 16S rRNA gene database and workbench compatible with ARB. *Appl. Environ. Microbiol.* 72, 5069-5072.

Edgar, R.C. (2004). MUSCLE: multiple sequence alignment with high accuracy and high throughput. *Nucleic Acids Res.* 32, 1792-1797.

Goujon, M. et al. (2010). A new bioinformatics analysis tools framework at EMBL-EBI. *Nucleic Acids Res.* 38, W695-W699. doi: 10.1093/nar/gkq313

Huerta-Cepas, J., Serra, F., and Bork, P. (2016). ETE 3: Reconstruction, analysis and visualization of phylogenomic data. *Mol. Biol. Evol.* 33, 1635-8. doi: 10.1093/molbev/msw046

Katoh, K., and Standley, D.M. (2013). MAFFT Multiple Sequence Alignment Software Version 7: Improvements in Performance and Usability. *Mol. Biol. Evol.* 30, 772-780.

Kõljalg, U. et al. (2013). Towards a unified paradigm for sequence-based identification of fungi. *Mol. Ecol.* 22, 5271-5277.

Kozich, J.J., Westcott, S.L., Baxter, N.T., Highlander, S.K., and Schloss, P.D. (2013). Development of a dual-index sequencing strategy and curation pipeline for analyzing amplicon sequence data on the MiSeq Illumina sequencing platform. *Appl. Environ. Microbiol.* 79, 5112-5120.

Larkin, M.A. et al. (2007). Clustal W and Clustal X version 2.0. *Bioinformatics.* 23, 2947-2948.

Munoz-Cadavid, C. et al. (2010). Improving molecular detection of fungal DNA in formalin-fixed paraffin-embedded tissues: comparison of five tissue DNA extraction methods using panfungal PCR. *J. Clin. Microbiol.* 48, 2147-2153.

Olsen, G. *"Newick's 8:45" Tree Format Standard*. 1990. Available: http://evolution.genetics.washington.edu/phylip/newick_doc.html, Accessed 2016.

R Development Core Team (2008). *R: A language and environment for statistical computing.* Vienna, Austria, R Foundation for Statistical Computing.

Sayers, E. W. et al. (2009). Database resources of the National Center for Biotechnology Information. *Nucleic Acids Res.* 37, D5-15. doi: 10.1093/nar/gkn741

Schloss, P.D. et al. (2009). Introducing mothur: open-source, platform-independent, community-supported software for describing and comparing microbial communities. *Appl. Environ. Microbiol.* 75, 7537-7541.
