# Supplementary material for: Critical Issues in Mycobiota Analysis
Source: Front Microbiol. 2017 Feb 14;8:180. doi: 10.3389/fmicb.2017.00180 (PMC5306204; doi:10.3389/fmicb.2017.00180)
Supplement: Supplementary file 7 [file DataSheet2.ZIP › supplementary_data_sheet_S2C.html]

```
Reference sequence (1): gi|698379407|gb|KM576414.1|_Hydnum-sp.-LM1961
Identities normalised by aligned length.
Colored by: identity + property
```

|  |
| --- |
| ```                                                                              1 [        .         .         .         .         :         .         .         . 80  1 gi|698379407|gb|KM576414.1|_Hydnum-sp.-LM1961                     100.0%     ----------------------------------------------------------------CATTATTGAATATTAC     2 gi|166178871|gb|EU379241.1|_Spizellomyces-punctatus-isolate-SW001  45.3%     -------------------------------TTAGTCCCTTATCAAAAACCAACCCTGTGTGAACTGTTTTGTCTCTGGC     3 gi|597709871|gb|KJ130481.1|_Piromyces-sp.-AIB01-11                 29.6%     CTACCGATTGAATG---------------GCTTAGCTACTACCGATTCAAATTTAGGGAATAGGCTTTCATAAATAT---     4 gi|597439552|dbj|AB916546.1|_Mucor-circinelloides                  39.2%     ------------------------------------TTTCCCGTAGGGGGCCCTGCGGAAGGATCATTAAATAAT-----     5 gi|56791302|gb|AY842567.1|_Glomus-versiforme                       38.2%     ---------------------------------------TCCGTAGGTGAACCTGCGGAAGGATCATTAAAA--------     6 gi|906358325|dbj|LC068778.1|_Aspergillus-flavus                    36.4%     ---------------------------------------------GGGGGGTATGCGGAAGGATCATTACCGAGTGTAGG     7 gi|62866693|gb|AY997087.1|_Rozella-allomycis                       37.5%     TAGTTGGCTCTAAACCGTTGGGTTATCGTGCTTGCACTTTAACTTGACAAATCTACTATGTGAACTTTATAATTAACGTG       consensus/100%                                                               ................................................................C..T............       consensus/90%                                                                ................................................................C..T............       consensus/80%                                                                ..............               ...................r....y...r.r.rr.C.TT...r........       consensus/70%                                                                                               ........y.....rr.rr.y.TrC.G.r.GA.C.TTA..r..T.y...                                                                                  81          .         1         .         .         .         .         :         . 160 1 gi|698379407|gb|KM576414.1|_Hydnum-sp.-LM1961                     100.0%     T--GGGGGCTGGATGCTGGCAGTCTATGATTGCATGTGCTCGCTCTCTTTAAT-------CTACTTACACATGTGCACC-     2 gi|166178871|gb|EU379241.1|_Spizellomyces-punctatus-isolate-SW001  45.3%     G--GCTGGGTAGAGATATACTTTGTGTAACCCTATCTGTCAGCTTGGTCTTATC----------TGAGCCGTGGACAACC     3 gi|597709871|gb|KJ130481.1|_Piromyces-sp.-AIB01-11                 29.6%     ---TTGTTGTTTGAAATAATTTTTTTTTAAAATTATTGATGTCTTTCCCTGTT----GAATTGGTTGGTCGTAAGACTAA     4 gi|597439552|dbj|AB916546.1|_Mucor-circinelloides                  39.2%     -------------CAATAATTTTGGCTTGTCCATTATTATCTATTTACTG--------------TGAACTGTATTATTAC     5 gi|56791302|gb|AY842567.1|_Glomus-versiforme                       38.2%     ------------------ACTTTTATCCGGGAATTCGTTTCGTTTTCCCGGAT---------------------------     6 gi|906358325|dbj|LC068778.1|_Aspergillus-flavus                    36.4%     G--TTCCTAGCGAGCCCAACCTCCCACCCGTGTTTACTGTACCTTAGTTGCTTCGGCGGGCCCGCCATTCATGGCCGCCG     7 gi|62866693|gb|AY997087.1|_Rozella-allomycis                       37.5%     GTCTCTGCAGTGTGGTCAAAGCTCTACCAAGGTTACTTATAGCTTTGGTATCT-------TTGGCCACACGTAT------       consensus/100%                                                               ..................r...y...y............y...Ty...y...............................       consensus/90%                                                                ..................r...y...y............y...Ty...y...............................       consensus/80%                                                                .  ...............Ay.yT...y.r.......y..T..yTT..yy...T.   .......y.r..yrTr.......       consensus/70%                                                                .  ...........r.yrAyyTTyy.yyr..ryTT.T..T..CTTT.yy...T       ....y.A..CrTr.......                                                                                 161          .         .         .         2         .         .         .         . 240 1 gi|698379407|gb|KM576414.1|_Hydnum-sp.-LM1961                     100.0%     ---------------------TCAATTTTGAAGA-------------------------CGAGGTAAAGCTTGTCTTTGG     2 gi|166178871|gb|EU379241.1|_Spizellomyces-punctatus-isolate-SW001  45.3%     CG-----CCAAACAATACTGAACAAACTTAGCTA-------------------------ATTTTTTCTGTCTAATATCTG     3 gi|597709871|gb|KJ130481.1|_Piromyces-sp.-AIB01-11                 29.6%     GTGATTCTCAGGTGATAGTCAATAATTTTTAAAA--------------------------AGACCTTTTTTTAAATTAAA     4 gi|597439552|dbj|AB916546.1|_Mucor-circinelloides                  39.2%     TTGACGCTTGAGGGATGCTCCACTGCTATAAGGATAGGCGGTGGGGATGTTAACCGAGTCATAGTCAAGCTTAGGCTTGG     5 gi|56791302|gb|AY842567.1|_Glomus-versiforme                       38.2%     -------------------------------------------------------------TATTTGTATTCAAATCCCA     6 gi|906358325|dbj|LC068778.1|_Aspergillus-flavus                    36.4%     GGGGCTCTCAGCCCCGGGCCCGCGCCCGCCGGAG--------------------------ACACCACGAACTCTGTCTGA     7 gi|62866693|gb|AY997087.1|_Rozella-allomycis                       37.5%     ---------------------GCGAGCACTGGGG-----------------------------CTTTTGCTAAACAATTA       consensus/100%                                                               ................................................................y.....y........r       consensus/90%                                                                ................................................................y.....y........r       consensus/80%                                                                ......................y...y.y.r..r                         .....y...ryyyr...yy.r       consensus/70%                                                                .....................rCrr.y.y.rrrr                         ...ryTy..ryTTAr.yyy.r                                                                                 241          :         .         .         .         .         3         .         . 320 1 gi|698379407|gb|KM576414.1|_Hydnum-sp.-LM1961                     100.0%     GATTTTATAAACTCTTACTCGATGTA---ATGAATGTTTTTGTCTGCCGAAAGGCAAAATTT---AATACAACTTTTAAC     2 gi|166178871|gb|EU379241.1|_Spizellomyces-punctatus-isolate-SW001  45.3%     ATTTGTATAAAT-----------------------------------------------GTA---CAAACAACTTTTGGC     3 gi|597709871|gb|KJ130481.1|_Piromyces-sp.-AIB01-11                 29.6%     CTTTTTGTATTCATTTGTCT---------AAAATTATTTTTATAATATA----------------AAAACAACTTTTGAG     4 gi|597439552|dbj|AB916546.1|_Mucor-circinelloides                  39.2%     TATCCTATTATTATTTACCAAAAGAATTCAGAATTAATATTGTAACATAGACCTAAAAAATCTATAAAACAACTTTTAAC     5 gi|56791302|gb|AY842567.1|_Glomus-versiforme                       38.2%     CTCTTTATAAAT-----------------AATATCAATTATATAAAACAAATATAAAA-------AAGAAAACTTTCAAC     6 gi|906358325|dbj|LC068778.1|_Aspergillus-flavus                    36.4%     TCTAGTGAAGTC-----------------TGAGTTGATTGTATCGCAATCA--------------GTTAAAACTTTCAAC     7 gi|62866693|gb|AY997087.1|_Rozella-allomycis                       37.5%     ATTCCTTTATTGCCT--------------AAAAACGATTATTTATAATCAC--------------AACACAACCTTTAAC       consensus/100%                                                               ..y..T..............................................................A.AACyTTyrr.       consensus/90%                                                                ..y..T..............................................................A.AACyTTyrr.       consensus/80%                                                                ..Ty.TrTA..y..............   ...r.yr.T..T.T...................   rA.A.AACTTTyrAC       consensus/70%                                                                .yTyyTrTAr.y........         ArrA.yr.TT.TrT...Ay.......... ...   AA.ACAACTTTTAAC                                                                                 321          .         .         :         .         .         .         .         4 400 1 gi|698379407|gb|KM576414.1|_Hydnum-sp.-LM1961                     100.0%     AACGGATCTCTTGGCTCTCGCATCGATGAAGAACGCAGCGAAATGCGATAAGTAATGTGAATTGCAGAATTCAGTGAATC     2 gi|166178871|gb|EU379241.1|_Spizellomyces-punctatus-isolate-SW001  45.3%     AACGGATCTCTTGGCTCTCGCAACGATGAAGAACGCAGCGAAATGCGATAAGTAGTGTGAATTGCAGAATTCAGTGAATC     3 gi|597709871|gb|KJ130481.1|_Piromyces-sp.-AIB01-11                 29.6%     AATGGATCTCTTGGTTCTCGCAACGATGAAGAACGCAGCAAAATGCGATAAGTAGTGTGAATTGCA-GAATACGTGAATC     4 gi|597439552|dbj|AB916546.1|_Mucor-circinelloides                  39.2%     AACGGATCTCTTGGTTCTCGCATCGATGAAGAACGTAGCAAAGTGCGATAACTAGTGTGAATTGCA-TATTCAGTGAATC     5 gi|56791302|gb|AY842567.1|_Glomus-versiforme                       38.2%     AACGGATCTCTTGGCTCTCGCATCGATGAAGAACGCAGCGAAATGCGATACGTAATGTGAATTGCAGAATTCCGTGAATC     6 gi|906358325|dbj|LC068778.1|_Aspergillus-flavus                    36.4%     AATGGATCTCTTGGTTCCGGCATCGATGAAGAACGCAGCGAAATGCGATAACTAGTGTGAATTGCAGAATTCCGTGAATC     7 gi|62866693|gb|AY997087.1|_Rozella-allomycis                       37.5%     AATGGATCTCTTGGCTCCTGCAACGATGAAGAACGCAGCGAAATGCGATATGTAATGTGAATTGCATGCATTCGTGAATC       consensus/100%                                                               AAyGGATCTCTTGGyTCy.GCA.CGATGAAGAACGyAGCrAArTGCGATA..TArTGTGAATTGCA....T..GTGAATC       consensus/90%                                                                AAyGGATCTCTTGGyTCy.GCA.CGATGAAGAACGyAGCrAArTGCGATA..TArTGTGAATTGCA....T..GTGAATC       consensus/80%                                                                AAyGGATCTCTTGGyTCyyGCA.CGATGAAGAACGCAGCrAAATGCGATA..TArTGTGAATTGCA.rA.Ty.GTGAATC       consensus/70%                                                                AAyGGATCTCTTGGyTCTCGCA.CGATGAAGAACGCAGCGAAATGCGATAAGTArTGTGAATTGCA.rATTC.GTGAATC                                                                                 401          .         .         .         .         :         .         .         . 480 1 gi|698379407|gb|KM576414.1|_Hydnum-sp.-LM1961                     100.0%     ATCGAATCTTTGAACGCACCTTGCGCTCTCTGGTATTCCGGAGA--GTACGCCTGTTCGAGTGTCA--------------     2 gi|166178871|gb|EU379241.1|_Spizellomyces-punctatus-isolate-SW001  45.3%     ATCGAATCTTTGAACGCACATTGCGCTCCCTGGTATTCCGGGGA--GCATGCCCGTTTCAGAATCAT-------------     3 gi|597709871|gb|KJ130481.1|_Piromyces-sp.-AIB01-11                 29.6%     ATCGAATCTTCGAACGCATATTGCACTTTTCTAGTTTACTAGAATTGTATGTCTGTTTGAGCAGTA--------------     4 gi|597439552|dbj|AB916546.1|_Mucor-circinelloides                  39.2%     ATCGAGTCTTTGAACGCAACTTGCGCTCATTGGTATTCCAATGA--GCACGCCTGTTTCAGTATCA--------------     5 gi|56791302|gb|AY842567.1|_Glomus-versiforme                       38.2%     ATCGAATCTTTGAACACAAATTGTACTTTTCAGTAATCTGGAAA--GTATGCTTGGTTGAGGGTCA--------------     6 gi|906358325|dbj|LC068778.1|_Aspergillus-flavus                    36.4%     ATCGAGTCTTTGAACGCACATTGCGCCCCCTGGTATTCCGGGGG--GCATGCCTGTCCGAGCGTCATTGCTGCC------     7 gi|62866693|gb|AY997087.1|_Rozella-allomycis                       37.5%     ATCGAATCTTTGAACGCATATTGCACTTCCTGGTATTCCGGGAA--GTATGTCTGTTTGAGTATCATATCTCTCTCTCTA       consensus/100%                                                               ATCGArTCTTyGAACrCA..TTGyrCyy.yy.r...T.y.r.rr..GyAyGyyyG.yy.AG.r.yA..............       consensus/90%                                                                ATCGArTCTTyGAACrCA..TTGyrCyy.yy.r...T.y.r.rr..GyAyGyyyG.yy.AG.r.yA..............       consensus/80%                                                                ATCGArTCTTTGAACGCA..TTGCrCTyyyyrGTATTCCrrrrA  GyAyGyCTGTTy.AG.rTCA........             consensus/70%                                                                ATCGAATCTTTGAACGCAyATTGCrCTyyyTGGTATTCCGGrrA  GyATGCCTGTTTGAGyrTCA.                                                                                              481          .         5         .         .         .         .         :         . 560 1 gi|698379407|gb|KM576414.1|_Hydnum-sp.-LM1961                     100.0%     -----------TGAAACTCTCAGGCAGAGATAGCTTTG------------------TTGCTGTTTTTGTTTGGATTTGGA     2 gi|166178871|gb|EU379241.1|_Spizellomyces-punctatus-isolate-SW001  45.3%     ----------TTTAATATCTCACCAAGTTATGGGTGCA------------------AGCTCATAACCAGGTGGAATTTGG     3 gi|597709871|gb|KJ130481.1|_Piromyces-sp.-AIB01-11                 29.6%     -----------------AAATATTC-------------------------------------------------------     4 gi|597439552|dbj|AB916546.1|_Mucor-circinelloides                  39.2%     -------------AAACAAACCCTC------------------------TATCCAACATTTTGTTGAATAGGAATACTGA     5 gi|56791302|gb|AY842567.1|_Glomus-versiforme                       38.2%     -----------TTAAAATAACATTCGTGAATTTTTTCGCGGATTTGAGTTTTCCAGTATTCATTATAAAATAAATGTTGG     6 gi|906358325|dbj|LC068778.1|_Aspergillus-flavus                    36.4%     ----------CATCAAGCACGGCTTGTGTGTTGGGTCG-----------TCGTCCCCTCTCCGGGGGGGACGGGCCCCAA     7 gi|62866693|gb|AY997087.1|_Rozella-allomycis                       37.5%     CTATACTTGTTATAGAAGAGGACTTGAGCGTC------------------------CATTCTTCACCGGACGTCGCTTTA       consensus/100%                                                               ................................................................................       consensus/90%                                                                ................................................................................       consensus/80%                                                                          ....r......ryyy.............           ..........yy......r...r....y..r       consensus/70%                                                                          ...AAA..A.yAyTyr...rT.......           .......y.yTy......r.ryGrr..yyrr                                                                                 561          .         .         .         6         .         .         .         . 640 1 gi|698379407|gb|KM576414.1|_Hydnum-sp.-LM1961                     100.0%     CTTTGCTGTGCC--AATGCGGC--------------TGGTCTTAAATGTATTAGCTGGTCCTAATATGGGGGTTTTGGT-     2 gi|166178871|gb|EU379241.1|_Spizellomyces-punctatus-isolate-SW001  45.3%     ATGTTTTGTCTC--TGTACAATC----ATCTTAAAATGATCGTATGTGCTTGAGTGTGCTCTGGCAGTTGGGTACCAACG     3 gi|597709871|gb|KJ130481.1|_Piromyces-sp.-AIB01-11                 29.6%     --------------------------------------------------------------------------------     4 gi|597439552|dbj|AB916546.1|_Mucor-circinelloides                  39.2%     GAGTCTCTTGATCTATTCTGATCTCGAACCTCTTGAAATGTACAAAGGCCTGATCTTGTTTGAATGCCTGAACTTTTTTT     5 gi|56791302|gb|AY842567.1|_Glomus-versiforme                       38.2%     TAACTTTAAAATTATTTATAACTTGGTACAAGCTGAAAACGTGCTATATGTGTGGTTCGCTGACAACTTGTCCATCTTTA     6 gi|906358325|dbj|LC068778.1|_Aspergillus-flavus                    36.4%     AGGCAGCGG--------------CGGCACCGCGTCCGATCCTCGAGCGTATGGGGCTTTGTCA-----------------     7 gi|62866693|gb|AY997087.1|_Rozella-allomycis                       37.5%     AATTTATGGGGC---GTGCAGCTTAACACTGCCTAGTAGACTTACAGGCTTACCAGTCGCTAACAAACAGCGTTTGCCCT       consensus/100%                                                               ................................................................................       consensus/90%                                                                ................................................................................       consensus/80%                                                                ...y..y..............................r.......r.ry.T.........y.r.................       consensus/70%                                                                ...y..yr...y  ..T.yrry.....Ay........r..y.yr.r.Gy.Tr....T..yy.A..r...G..y.y...y.                                                                                 641          :         .         .         .         .         7         .         . 720 1 gi|698379407|gb|KM576414.1|_Hydnum-sp.-LM1961                     100.0%     ---------------TCTACTCAGCGTGATAATTATCTGACGCTGAGGAC---AGTCTTAGGACTG-------------G     2 gi|166178871|gb|EU379241.1|_Spizellomyces-punctatus-isolate-SW001  45.3%     TGCAACAGATTTTA-TCTTTTCGCTTTGGTATTTAATTGAAATCCGGTACCTGAAGCATATATTTGCTGGCTTACAGATG     3 gi|597709871|gb|KJ130481.1|_Piromyces-sp.-AIB01-11                 29.6%     ------------------------------------------TCATAA-------AAACATTTTTGTTCA----------     4 gi|597439552|dbj|AB916546.1|_Mucor-circinelloides                  39.2%     TAATATAAAGAGAAGCTCTTGCGGTAAACTGTGCTGGGGCCTCCCAAA-------TAATACTTTTTTTAAA----ATTGA     5 gi|56791302|gb|AY842567.1|_Glomus-versiforme                       38.2%     TATATTA--------TGCGCGTGCTTGGCTTTTTAAAGTTCTGTGCGA-------GTATATATTTTTTTA-------TGA     6 gi|906358325|dbj|LC068778.1|_Aspergillus-flavus                    36.4%     ---------------CCCGCTCTGTAGGCCCGGCCGGCGCTTGCCGAACG---CAAATCAATCTTTTCCAG----GTTGA     7 gi|62866693|gb|AY997087.1|_Rozella-allomycis                       37.5%     TATCAT---------GCCATTCAGTGTGGTAAGTAAGCAGGCGTTGGATG---G-TAATGTAGAGTTTGA-------CGA       consensus/100%                                                               ...........................................y..r...........yr....................       consensus/90%                                                                ...........................................y..r...........yr....................       consensus/80%                                                                .............. ..y.y.y..y..r.y...y.........y..rr..   .....yA...yT.yy.r.    ....r       consensus/70%                                                                .......        yyy.y.Cr.T..G.T...y.r..r....y.rrA..   ....ATAy.yTT.TT.A.    ....r                                                                                 721          .         .         :         .         .         .   ] 784 1 gi|698379407|gb|KM576414.1|_Hydnum-sp.-LM1961                     100.0%     CCAGAGCTTATGTTTGGATTGCTTC---------------------------------------     2 gi|166178871|gb|EU379241.1|_Spizellomyces-punctatus-isolate-SW001  45.3%     TTATGCATAATGCATGTATAGCTTCTGAT----------AACTTGGTCTGAAA-----------     3 gi|597709871|gb|KJ130481.1|_Piromyces-sp.-AIB01-11                 29.6%     ----------------------CACTGGTTTTAAAAACAACTTTTGACAATGGATCTAACGCAG     4 gi|597439552|dbj|AB916546.1|_Mucor-circinelloides                  39.2%     TCTGAAATCAGGCGGGATTACCCGCTGAACTTAAGCATATCAATAGCCGGAGG-----------     5 gi|56791302|gb|AY842567.1|_Glomus-versiforme                       38.2%     CCTCAGCTCAAGCAAGAAAACCCGCTGAACTTAAGCATATCAATAAGCGGAGGA----------     6 gi|906358325|dbj|LC068778.1|_Aspergillus-flavus                    36.4%     CCTCGGATCAGGTAGGGATACCCGCTGAACTTAAGCATATCAATAACCGGAGGAAGTT------     7 gi|62866693|gb|AY997087.1|_Rozella-allomycis                       37.5%     ACGGTTAGCAAGC-----TGTCCTTTGGCTTTTAAAGTCAGAGTATGATTAAG-----------       consensus/100%                                                               ......................y.y.......................................       consensus/90%                                                                ......................y.y.......................................       consensus/80%                                                                .y.......A.Gy........Cy.CTGr...............T.......rr.....             consensus/70%                                                                yC..r..TyA.Gy..G..Tr.CC.CTGr.yTT.Ar.ry.....Trr.C.rArG. ``` |
