# Supplementary material for: Critical Issues in Mycobiota Analysis
Source: Front Microbiol. 2017 Feb 14;8:180. doi: 10.3389/fmicb.2017.00180 (PMC5306204; doi:10.3389/fmicb.2017.00180)
Supplement: Supplementary file 7 [file DataSheet2.ZIP › supplementary_data_sheet_S2A.html]

```
Reference sequence (1): J01695.2
Identities normalised by aligned length.
Colored by: identity + property
```

|  |
| --- |
| ```                                      1 [        .         .         .         .         :         .         .         . 80   1 J01695.2                 100.0%      --AAATTGAAGAGTTTGATCATGGCTCAGATTGAACGCTGGCGGCAGGCCTAACACATGCAAGTCGAACGGTAACAGGAA      2 160267|DQ229066.1         87.7%      ----------------------------------------------------ACACATGCAG--TCGACGGTAGCAGGAA      3 101027|NC_005973.1        72.0%      ATCTCATGGAGAGTTCGATCCTGGCTCAGGATGAACGCTGGCGGCATGCTTAACACATGCAAGTCGAACGGGAAGT----      4 268298|NC_010337.2        75.3%      ------TGGAGAGTTTGATCCTGGCTCAGGACGAACGCTGGCGGCATGCCTAACACATGCAAGTCGAACGGAGAGCTGAA      5 592884|NR_029335.1        73.5%      --------------------------------------------------------------------------------      6 584703|NR_028933.1        72.8%      ----------GAGTTTGATCCTGGCTCAGGATGAACGCTGACAGAATGCTTAACACATGCAAGTCAACTCGAGTCT----      7 588215|EU703281.1         72.4%      ----------------------------------------------------------GCAAGTCGAACGGAGTATTTTT      8 471149|NZ_ACUZ01000110.1  69.2%      -TACAATGGAGAGTTTGATCCTGGCTCAGGATGAACGCTGGCTACAGGCTTAACACATGCAAGTCGAGGGGAAACGACGG        consensus/100%                       ................................................................................        consensus/90%                        ................................................................................        consensus/80%                         .........................................................GCAr..y.r...G.r.......        consensus/70%                          ..................................................ACACATGCAAGTCrAryGG.r.......                                           81          .         1         .         .         .         .         :         . 160  1 J01695.2                 100.0%      GA-AGCTTGCTT-CTTTGCTGACGAGTGGCGGACGGGTGAGTAATGTCTGGG-AAACTGCCTGATGGAGGGGGATAACTA      2 160267|DQ229066.1         87.7%      GA-AGCTTGCTT-CTTTGCTGACGAGTGGCGGACGGGTGAGTAATGCTTGGG-AATCTGGCTTATGGAGGGGGATAACTA      3 101027|NC_005973.1        72.0%      ---------------GGTGTTTCCAGTGGCGAACGGGTGAGTAACGCGTAAG-AACCTGCCCTTGGGAGGGGAACAACAA      4 268298|NC_010337.2        75.3%      G-----TTTCGA-TGGAGGCTCTTAGTGGCGGACGGGTGAGTAACGCGTGGATAACCTGCCTGAGAGTGGGGGATAACAG      5 592884|NR_029335.1        73.5%      ----------------------GGAGTGGCGAACGGGTGAGTAATACATAAGCAATCTGCCCATCGGCCTGGGATAACAG      6 584703|NR_028933.1        72.8%      ---------------TCGGACTTGGGTGGCGGACGGGTGAGTAACGCGTAAAGAACTTGCCTCTTAGTCTGGGACAACAT      7 588215|EU703281.1         72.4%      AG-AGCTTGCTT-TGAAAATGCCTAGTGGCGAACGGGTGAGTAGCACGTGAGTTACCTGCCCCGAAGCGGGGGATAGCCC      8 471149|NZ_ACUZ01000110.1  69.2%      GGAAGCTTGCTTCCTTGGGCGTCGACCGGCGCACGGGTGAGTAACGCGTATCCAACCTGCCTCTGACTGAGGGATAACCC        consensus/100%                       ........................r.yGGCG.ACGGGTGAGTAryry.Tr....A.yTG.Cy...r....GGrAyArC..        consensus/90%                        ........................r.yGGCG.ACGGGTGAGTAryry.Tr....A.yTG.Cy...r....GGrAyArC..        consensus/80%                        .. ......... .........y.AGTGGCGrACGGGTGAGTAAyrC.Trrr.AAyCTGCCy...rG...GGGAyAAC..        consensus/70%                        .. ......... ....r.y..y.AGTGGCGrACGGGTGAGTAAyGCrTrrr.AAyCTGCCy...rG.GrGGGATAAC..                                          161          .         .         .         2         .         .         .         . 240  1 J01695.2                 100.0%      CTGGAAACGGTAGCTAATACCGCATAA-----------------CGTCGCAAGACCAAAGAGGGGGACCTTCGGGCCTCT      2 160267|DQ229066.1         87.7%      CGGGAAACTGTAGCTAATACCGCGTAG-----------------TATCGAGAGATGAAAGACTGGGACCGCAAGGCCAGT      3 101027|NC_005973.1        72.0%      CTGGAAACGGTTGCTAATACCCCGTAG------------------GCTGAGGAGCAAAA------------GGAGAAATC      4 268298|NC_010337.2        75.3%      CCCGAAAGGGCTGCTAATACCGCATAACGTTCCTGAAAGACATCTTTCGGGAACCAAA-------------GGAGCAATC      5 592884|NR_029335.1        73.5%      TTGGAAACGACTGCTAATACCGGATAG-GTGATGAAGAGGCATCTCTCGATCATTAAAGT----------TGGGATACAA      6 584703|NR_028933.1        72.8%      CTGGAAACGGATGCTAATACCGGATATTATGCT---------TTTTTCGCATGGAGAAAGCATGA-----AAGCTACATG      7 588215|EU703281.1         72.4%      GAAGAAATTCGGATTAATACCCCATGT-----------------GATCGAAAGATTAAAGGCGG------CGTAAGCTGT      8 471149|NZ_ACUZ01000110.1  69.2%      GTCGAAAGTCGGCCTAATACCTCATGGCATCGT-----------CTGCGGGCATCCAACGACGATTA---AAGATTTCAT        consensus/100%                       ...GAAA......yTAATACC..rTr.....................yG...r...AA......................        consensus/90%                        ...GAAA......yTAATACC..rTr.....................yG...r...AA......................        consensus/80%                        ...GAAA.....rCTAATACC..rTr....................yCG.r.r.y.AA.............rrr......        consensus/70%                        yyrGAAAy.r..GCTAATACC.CATAr......         ..y.TCGrr.r.y.AAr........   .rGrr....y                                          241          :         .         .         .         .         3         .         . 320  1 J01695.2                 100.0%      TGCCATCGGATGTGCCCAGATGGGATTAGCTAGTAGGTGGGGTAACGGCTCACCTAGGCGACGATCCCTAGCTGGTCTGA      2 160267|DQ229066.1         87.7%      TGCCATGAGATGAGCCCAAGTGGGATTAGGTAGTTGGTGGGGTAAAGGCCTACCAAGCCGACGATCTCTAGCTGGTCTGA      3 101027|NC_005973.1        72.0%      CGCCCAAGGAGGGGCTCGCGTCTGATTAGCTAGTTGGTGAGGCAATAGCTTACCAAGGCGATGATCAGTAGCTGGTCCGA      4 268298|NC_010337.2        75.3%      CGCTGTCAGATGGGTCCGCGTCCGATTAGCTAGTTGGCGGGGTAAGAGCCCACCAAGGCGACGATCGGTAGCCGGCCTGA      5 592884|NR_029335.1        73.5%      CACGGATGGATGAGCTTATGGCGTATTAGCTAGTAGGTGAGGTAACGGCTCACCTAGGCGATGATACGTAGCCGACCTGA      6 584703|NR_028933.1        72.8%      CGCTAAGAGAGAGCTTTGCGTCCCATTAGCTCGTTGGTGAGGTAACGGCTCACCAAGGCAATGATGGGTACCCGGCCTGA      7 588215|EU703281.1         72.4%      CGCTTCGGGATGGGCTCGCGGCCTATCAGCTTGTTGGTGAGGTAACGGCTCACCAAGGCGATGACGGGTAGCTGATCTGA      8 471149|NZ_ACUZ01000110.1  69.2%      CGGTCAGGGATGGGGATGCGTCTGATTAGCTTGTTGGCGGGGTAACGGCCCACCAAGGCTACGATCAGTAGGGGTTCTGA        consensus/100%                       yr.....rGA.r....yr.r....ATyAG.T.GT.GGyGrGGyAA.rGCyyACC.AG.C.AyGAy...TA...G.yCyGA        consensus/90%                        yr.....rGA.r....yr.r....ATyAG.T.GT.GGyGrGGyAA.rGCyyACC.AG.C.AyGAy...TA...G.yCyGA        consensus/80%                        yGCy...rGA.GrGyyyr.G....ATTAGCT.GT.GGyGrGGTAA.rGCyyACC.AGGCrAyGAT...TAGCyGryCTGA        consensus/70%                        CGCy...rGATGrGyyyryGTC..ATTAGCT.GTTGGTGrGGTAAyGGCyCACCAAGGCGAyGAT..GTAGCyGryCTGA                                          321          .         .         :         .         .         .         .         4 400  1 J01695.2                 100.0%      GAGGATGACCAGCCACACTGGAACTGAGACACGGTCCAGACTCCTACGGGAGGCAGCAGTGGGGAATATTGCACAATGGG      2 160267|DQ229066.1         87.7%      GAGGATGACCAGCCACACTGGAACTGAGACACGGTCCAGACTCCTACGGGAGGCAGCAGTGGGGAATATTGCACAATGGG      3 101027|NC_005973.1        72.0%      GAGGATGATCAGCCACACTGGGACTGAGACACGGCCCAGACTCCTACGGGAGGCAGCAGTGGGGAATTTTCCGCAATGGG      4 268298|NC_010337.2        75.3%      GAGGGTGAACGGCCACACTGGGACTGAGACACGGCCCAGACTCCTACGGGAGGCAGCAGTGGGGAATCTTCCGCAATGGG      5 592884|NR_029335.1        73.5%      GAGGGTGACCGGCCACATTGGGACTGAGACACGGCCCAAACTCCTACGGGAGGCAGCAGTAGGGAATTTTCGGCAATGGG      6 584703|NR_028933.1        72.8%      GAGGGTGAACGGCCACAAGGGGACTGAGACACGGCCCTTACTCCTACGGGAGGCAGCAGTGGGGAATATTGGACAATGGA      7 588215|EU703281.1         72.4%      GAGGATGATCAGTCACACTGGAACTGAGACACGGTCCAGACACCTACGGGTGGCAGCAGTCGAGAATCATTCGCAATGGG      8 471149|NZ_ACUZ01000110.1  69.2%      GAGGAAGGTCCCCCACATTGGAACTGAGACACGGTCCAAACTCCTACGGGAGGCAGCAGTGAGGAATATTGGTCAATGGG        consensus/100%                       GAGGr.Gr.C..yCACA..GGrACTGAGACACGGyCC..AC.CCTACGGG.GGCAGCAGT.rrGAAT..T...CAATGGr        consensus/90%                        GAGGr.Gr.C..yCACA..GGrACTGAGACACGGyCC..AC.CCTACGGG.GGCAGCAGT.rrGAAT..T...CAATGGr        consensus/80%                        GAGGrTGA.CrGCCACAyTGGrACTGAGACACGGyCCArACTCCTACGGGAGGCAGCAGTrGGGAAT.TT..rCAATGGG        consensus/70%                        GAGGrTGAyCrGCCACAyTGGrACTGAGACACGGyCCArACTCCTACGGGAGGCAGCAGTGGGGAAT.TT..rCAATGGG                                          401          .         .         .         .         :         .         .         . 480  1 J01695.2                 100.0%      -CGCAAGCCTGATGCAGCCATGCCGCGTGTATGAAGAAGGCCTT-CGGGTTGTAAAGTACTTTCAGCGGGGAGGAAGGGA      2 160267|DQ229066.1         87.7%      -GGGAACCCTGATGCAGCCATGCCGCGTGAATGAAGAAGGCCTT-CGGGTTGTAAAGTTCTTTCGGTGATGAGGAAGGGT      3 101027|NC_005973.1        72.0%      -CGAAAGCCTGACGGAGCAATGCCGCGTGGAGGTGGAAGGCCCA-CGGGTCGTCAACTTCTTTTCTCGGAGAAGAA----      4 268298|NC_010337.2        75.3%      -CGAAAGCCTGACGGAGCAATGCCGCGTGGGGGACGAAGGTCTT-CGGATTGTAAACCCTTGTCTTCAGGGAAGAA----      5 592884|NR_029335.1        73.5%      -CGAAAGCCTGACCGAGCAACGCCGCGTGAGTGAAGAAGGCCTT-CGGGTTGTAAAGCTCTGTTGTGAAGGAAGAACGGC      6 584703|NR_028933.1        72.8%      CCAAAAGTCTGATCCAGCAATTCTGTGTGCACGATGACGTTTTT-CGGAATGTAAAGTGCTTTCAGTCGGGAAGAA----      7 588215|EU703281.1         72.4%      -CGAAAGCCTGACGGTGCGACGCCGCGTGGAGGATGAAGGCCCT-AGGGTCGTAAACTCCTGTCATCAGGGAGTAATGGC      8 471149|NZ_ACUZ01000110.1  69.2%      -CGAGAGCCTGAACCAGCCAAGTAGCGTGCAGGAAGACGGCCCTATGGGTTGTAAACTGCTTTTATGCGGGGATAA----        consensus/100%                       ..r.rA.yCTGA....GC.A..y.GyGTG.r.G..GA.G.yyy...GGr.yGT.AA.y.yT.Ty....r.Grr.AA....        consensus/90%                        ..r.rA.yCTGA....GC.A..y.GyGTG.r.G..GA.G.yyy...GGr.yGT.AA.y.yT.Ty....r.Grr.AA....        consensus/80%                         CGrAAGCCTGAy..AGC.AyGCyGCGTG.r.GA.GA.GGyCyT yGGrTyGTAAA.y.CT.Ty....rrGAr.AA....        consensus/70%                         CGAAAGCCTGAy..AGC.AyGCCGCGTG.A.GA.GAAGGCCyT CGGGTTGTAAA.T.CT.Tyr.yrGGGArGAA....                                          481          .         5         .         .         .         .         :         . 560  1 J01695.2                 100.0%      GTAAAGTTAATACCTTT-GCTCATTGACGTTACCCGCAGAAGAAGCACCGGCTAACTCCGTGCCAGCAGCCGCGGTAATA      2 160267|DQ229066.1         87.7%      GGTGTTTTAATAGAGCA-CTACATTGACGTTAGTCACAGAAGAAGCACCGGCTAACTCCGTGCCAGCAGCCGCGGTAATA      3 101027|NC_005973.1        72.0%      --------------------ACAATGACGGTATCTGAGGAATAAGCATCGGCTAACTCTGTGCCAGCAGCCGCGGTAAGA      4 268298|NC_010337.2        75.3%      ---------------------GAATGACGGTACCTGAGGAGGAAGCCCCGGCTAACTACGTGCCAGCAGCCGCGGTAATA      5 592884|NR_029335.1        73.5%      TCATACAGGGAATGGTA-TGGGAGTGACGGTACTTTACCAGAAAGCCACGGCTAACTACGTGCCAGCAGCCGCGGTAATA      6 584703|NR_028933.1        72.8%      -------------------GAAAGTGACGGTACCGACAGAAGAAGCGACGGCTAAATACGTGCCAGCAGCCGCGGTAATA      7 588215|EU703281.1         72.4%      TCATATAGTAACTGATATGGGTCTTGATAGTACCTGAAGAGGAAGGGACGGCAAACTTCGTGCCAGCAGCCGCGGTAATA      8 471149|NZ_ACUZ01000110.1  69.2%      --AGTGAGGGACGTGTC-CTTCATTGCAGGTACCGCATGAATAAGGACCGGCTAATTCCGTGCCAGCAGCCGCGGTAATA        consensus/100%                       ........................TG..r.TA.y.....Ar.AAG...CGGC.AA.T.yGTGCCAGCAGCCGCGGTAA.A        consensus/90%                        ........................TG..r.TA.y.....Ar.AAG...CGGC.AA.T.yGTGCCAGCAGCCGCGGTAA.A        consensus/80%                        ................. ....A.TGAyG.TAyy....GAr.AAG...CGGCTAAyT.CGTGCCAGCAGCCGCGGTAATA        consensus/70%                        ................. ....A.TGACGGTACCyr.rGArrAAGCr.CGGCTAACT.CGTGCCAGCAGCCGCGGTAATA                                          561          .         .         .         6         .         .         .         . 640  1 J01695.2                 100.0%      CGGAGGGTGCAAGCGTTAATCGGAATTACTGGGCGTAAAGCGCACGCAGGCGGTTTGTTAAGTCAGATGTGAAATCCCCG      2 160267|DQ229066.1         87.7%      CGGAGGGTGCGAGCGTTAATCGGAATGACTGGGCGTAAAGGGCACGCAGGCGGTGACTTAAGTGAGATGTGAAAGCCCCG      3 101027|NC_005973.1        72.0%      CAGAGGATGCAAGCGTTATCCGGAATGATTGGGCGTAAAGCGTCTGTAGGTGGCTTTTCAAGTCCGCCGTCAAATCCCAG      4 268298|NC_010337.2        75.3%      CGTAGGGGGCGAGCGTTGTCCGGAATTACTGGGCGTAAAGCGCGTGCAGGCGGACCTTTAAGTCTGAGGTGAAAGACCGG      5 592884|NR_029335.1        73.5%      CGTAGGTGGCGAGCGTTATCCGGAATTATTGGGCGTAAAGGGTGCGCAGGCGGTTTGTTAAGTTTAAGGTGAAAGCGTGG      6 584703|NR_028933.1        72.8%      CGTATGTCGCAAGCGTTATCCGGATTTATTGGGCGTAAAGCGCGTCTAGGCGGCAAGGAAAGTCTGATGTGAAAATGCGG      7 588215|EU703281.1         72.4%      CGAAGGTCCCAAGCGTTGTTCGGATTTACTGGGCGTAAAGAGTCTGTAGGCGGTGGGGTAAGTCAGATGTGAAATCCCGG      8 471149|NZ_ACUZ01000110.1  69.2%      CGGAAGGTCCGGGCGTTATCCGGATTTATTGGGTTTAAAGGGAGCGTAGGCCGTGGATTAAGCGTGTTGTGAAATGCAGG        consensus/100%                       Cr.A.G...CrrGCGTTr.yCGGA.T.AyTGGGy.TAAAG.G..y.yAGGy.G......AAGy..r..GT.AAA.....G        consensus/90%                        Cr.A.G...CrrGCGTTr.yCGGA.T.AyTGGGy.TAAAG.G..y.yAGGy.G......AAGy..r..GT.AAA.....G        consensus/80%                        CG.ArG...CrAGCGTTr.yCGGA.T.AyTGGGCGTAAAG.Gy.yGyAGGCGGy....yAAGT..G..GTGAAA...y.G        consensus/70%                        CG.AGG.yGCrAGCGTTATyCGGA.TTAyTGGGCGTAAAG.GyryGyAGGCGGy...TTAAGTy.GAyGTGAAA.yCCrG                                          641          :         .         .         .         .         7         .         . 720  1 J01695.2                 100.0%      GGCTCAACCTGGGAACTGCATCTGATACTGGCAAGCTTGAGTCTCGTAGAGGGGGGTAGAATTCCAGGTGTAGCGGTGAA      2 160267|DQ229066.1         87.7%      AGCTTAACTTGGGAATTGCATTTCATACTGGGTTGCTAGAGTATTTTAGGGAGGGGTAGAATTCCACGTGTAGCGGTGAA      3 101027|NC_005973.1        72.0%      GGCTCAACCCTGGACAGGCGGTGGAAACTACCAAGCTGGAGTACGGTAGGGGCAGAGGGAATTTCCGGTGGAGCGGTGAA      4 268298|NC_010337.2        75.3%      AGCTCAACTCCGGGGCGGCCTTGGAAACTGGAGGTCTTGAGGGATGGAGAGGACAGTGGAATTCCCGGTGTAGCGGTGAA      5 592884|NR_029335.1        73.5%      GGCTTAACCCC-ATATAGCCTTAGAAACTGACAGACTAGAGTACAGGAGAGGGCAATGGAATTCCATGTGTAGCGGTAAA      6 584703|NR_028933.1        72.8%      GGCTCAACTCC-GTATTGCGTTGGAAACTGCCTTACTAGAGTACTGGAGAGGTAGGCGGAACTACAAGTGTAGAGGTGAA      7 588215|EU703281.1         72.4%      GGCTCAACCCCGGAACTGCATCCGATACTGCCTCACTTGAGGGTTGGAGGGGAGTCTGGAATTCTCGGTGTAGCAGTGAA      8 471149|NZ_ACUZ01000110.1  69.2%      TGCTCAACGTCTGCACTGCAGCGCGAACTGGTCCACTTGAGTGTGCGCAACGCAGGCGGAATTCGTCGTGTAGCGGTGAA        consensus/100%                       .GCTyAAC.y..r....GC..y..r.ACTr.....CT.GAG.......rr.r.....rGAAyT....GTG.AG.rGTrAA        consensus/90%                        .GCTyAAC.y..r....GC..y..r.ACTr.....CT.GAG.......rr.r.....rGAAyT....GTG.AG.rGTrAA        consensus/80%                        rGCTyAACyy..G.ry.GC..y..A.ACTG....rCT.GAG.ry...AGrGG..rryrGAATTyy..GTGTAGCGGTGAA        consensus/70%                        rGCTCAACyyy.G.Ay.GCrTy.GA.ACTG.y..rCT.GAGTry.G.AGrGG.rrryGGAATTCC..GTGTAGCGGTGAA                                          721          .         .         :         .         .         .         .         8 800  1 J01695.2                 100.0%      ATGCGTAGAGATCTGGAGGAATACCGGTGGCGAAGGCGGCCCCCTGGACGAAGACTGACGCTCAGGTGCGAAAGCGTGGG      2 160267|DQ229066.1         87.7%      ATGCGTAGAGATGTGGAGGAATACCGAAGGCGAAGGCAGCCCCTTGGGAAAATACTGACGCTCATGTGCGAAAGCGTGGG      3 101027|NC_005973.1        72.0%      ATGCATTGAGATCGGAAAGAACACCAACGGCGAAAGCACTCTGCTGGGCCGACACTGACACTGAGAGACGAAAGCTAGGG      4 268298|NC_010337.2        75.3%      ATGCGTAGATATCGGGAGGAACCCCAGTGGCGAAGGCGACTGTCTGGACATTACCTGACGCTGAGGCGCGAAAGCGTGGG      5 592884|NR_029335.1        73.5%      ATGCGTAGATATATGGAGGAACACCAGTGGCGAAGGCGGTTGCCTGGCCTGTAACTGACGCTCATGCACGAAAGCGTGGG      6 584703|NR_028933.1        72.8%      ATTCGTAGATATTTGTAGGAATGCCGATGGGGAAGCCAGCCTACTGGACAGATACTGACGCTAAAGCGCGAAAGCGTGGG      7 588215|EU703281.1         72.4%      ATGCGTAGATATCGAGAGGAACACTAGTGGCGAAGGCGAGACTCTGGACAACACCTGACGCTGAGAGACG-AAGCCAGGG      8 471149|NZ_ACUZ01000110.1  69.2%      ATGCTTAGATATGACGAAGAACTCCGATTGCGAAGGCAGCTTGCGGGAGCACAACTGACGCTGAAGCTCGAAAGTGCGGG        consensus/100%                       AT.C.T.GA.AT....ArGAAy.Cyrr..G.GAAr.Cr.....y.GG.......CTGACrCT.A.r..CG.AAGy..GGG        consensus/90%                        AT.C.T.GA.AT....ArGAAy.Cyrr..G.GAAr.Cr.....y.GG.......CTGACrCT.A.r..CG.AAGy..GGG        consensus/80%                        ATGCrTAGA.AT..rrArGAAy.CCrryGGCGAAGGCrryy..CTGGr..r...CTGACGCT.A.r.rCGAAAGC..GGG        consensus/70%                        ATGCGTAGA.AT..GGAGGAAyrCCrrTGGCGAAGGCrryyy.CTGGrC.r..ACTGACGCT.ArGyrCGAAAGCGyGGG                                          801          .         .         .         .         :         .         .         . 880  1 J01695.2                 100.0%      GAGCAAACAGGATTAGATACCCTGGTAGTCCACGCCGTAAACGATGTCGACTTGGAGGTTGTGCCCTTGAGG-CGTGGCT      2 160267|DQ229066.1         87.7%      GAGCAAACAGGATTAGATACCCTGGTAGTCCACGCTGTAAACGCTGTCGATTTGGGGATTGGGCTTAGAG---CTTGGTG      3 101027|NC_005973.1        72.0%      GAGCAAATGGGATTAGAGACCCCAGTAGTCCTAGCCGTAAACGATGGATACTAGGTGCTGTGCGACTCGACCCGTGCAGT      4 268298|NC_010337.2        75.3%      GAGCAAACAGGATTAGATACCCTGGTAGTCCACGCCGTAAACGATGAGTGCTAGGTGTTGGGGGTATCGACCCCTCCAGT      5 592884|NR_029335.1        73.5%      GAGCAAATAGGATTAGATACCCTAGTAGTCCACGCCGTAAACGATGAGAACTAAGTGTTGGGGAAA--------CTCAGT      6 584703|NR_028933.1        72.8%      TAGCAAACAGGATTAGATACCCTGGTAGTCCACGCTGTAAACGATGATTACTAGGTGTTGGGGGTCAAA----CCTCAGC      7 588215|EU703281.1         72.4%      GAGCGAAAAGGATTAGATACCCTTGTAGTCCTGGCAGTAAACGGTGCACGCTTGGTGTGGGAGGGTTCGACCCCTTCTGT      8 471149|NZ_ACUZ01000110.1  69.2%      TATCGAACAGGATTAGATACCCTGGTAGTCCGCACGGTAAACGATGGATGCCCGTTGTCAGGCTGTTTCA---GCCTGGT        consensus/100%                       .A.CrAA.rGGATTAGA.ACCCy.GTAGTCC..rC.GTAAACG.TG...ryy.r..G.......................        consensus/90%                        .A.CrAA.rGGATTAGA.ACCCy.GTAGTCC..rC.GTAAACG.TG...ryy.r..G.......................        consensus/80%                        .AGCrAAyAGGATTAGATACCCTrGTAGTCC..GC.GTAAACGrTG...rCT.GG.G.y.Gr............yy.r.y        consensus/70%                        GAGCAAAyAGGATTAGATACCCTrGTAGTCCrCGCyGTAAACGATG...rCT.GGTGyTrGGG..y..rr....yyyrGT                                          881          .         9         .         .         .         .         :         . 960  1 J01695.2                 100.0%      TCCGGAGCTAACGCGTTAAGTCGACCGCCTGGGGAGTACGGCCGCAAGGTTAAAACTCAAATGAATTGACGGGGGCCCGC      2 160267|DQ229066.1         87.7%      CCCGTAGCTAACGTGATAAATCGACCGCCTGGGGAGTACGGCCGCAAGGTTAAAACTCAAATGAATTGACGGGGGCCCGC      3 101027|NC_005973.1        72.0%      GCTGTAGCTAACGCGTTAAGTATCCCGCCTGGGGAGTACGTTCGCAAGAATGAAACTCAAAGGAATTGACGGGGGCCCGC      4 268298|NC_010337.2        75.3%      GCCGCAGTCAACACAATAAGCACTCCGCCTGGGGAGTACGGCCGCAAGGTTGAAACTCAAAGGAATTGACGGGGGCCCGC      5 592884|NR_029335.1        73.5%      GCTGCAGTTAACGCAATAAGTTCTCCGCCTGGGGAGTATGCACGCAAGTGTGAAACTCAAAGGAATTGACGGGGGCCCGC      6 584703|NR_028933.1        72.8%      GCCCAAGCTAACGCGATAAGTAATCCGCCTGGGGAGTACGTACGCAAGTATGAAACTCAAAGGAATTGACGGGGACCCGC      7 588215|EU703281.1         72.4%      GCCGGAGCTAACGCGTTAAGCGTGCCGCCTGGGGAGTACGGTCGCAAGATTAAAACTCAAAGAAATTGACGGGGACCCGC      8 471149|NZ_ACUZ01000110.1  69.2%      GACCAAGCGAAAGCATTAAGCATCCCACCTGGGGAGTACGCCGGCAACGGTGAAACTCAAAGGAATTGACGGGGGCCCGC        consensus/100%                       ..y..AGy.AA.ryr.TAAry...CCrCCTGGGGAGTAyG...GCAA...TrAAACTCAAA.rAATTGACGGGGrCCCGC        consensus/90%                        ..y..AGy.AA.ryr.TAAry...CCrCCTGGGGAGTAyG...GCAA...TrAAACTCAAA.rAATTGACGGGGrCCCGC        consensus/80%                        .Cy..AGyyAACGCr.TAAGy...CCGCCTGGGGAGTACG..CGCAAG..TrAAACTCAAA.GAATTGACGGGGrCCCGC        consensus/70%                        GCCG.AGCTAACGCr.TAAGy...CCGCCTGGGGAGTACG.yCGCAAGr.TrAAACTCAAAGGAATTGACGGGGGCCCGC                                          961          .         .         .         0         .         .         .         . 1040 1 J01695.2                 100.0%      ACAAGCGGTGGAGCATGTGGTTTAATTCGATGCAACGCGAAGAACCTTACCTGGTCTTGACATCCA-CGGAAGTTTTCAG      2 160267|DQ229066.1         87.7%      ACAAGCGGTGGAGCATGTGGTTTAATTCGATGCAACGCGAAGAACCTTACCTACTCTTGACATCCT-AAGAAGAACTCAG      3 101027|NC_005973.1        72.0%      ACAAGCGGTGGAGCATGTGGTTTAATTCGATGCAAAGCGAAGAACCTTACCAGGGCTTGACATGCC-GCGAATCCTCTTG      4 268298|NC_010337.2        75.3%      ACAAGCGGTGGAGCATGTGGTTTAATTCGACGCAACGCGAAGAACCTTACCAAGGCTTGACATCCT-CCGAACCTTGCAG      5 592884|NR_029335.1        73.5%      ACAAGCGGTGGAGTATGTGGTTTAATTCGACGCAACGCGAAGAACCTTACCAGGTCTTGACATCCCCTGCAAAGACATAG      6 584703|NR_028933.1        72.8%      ACAAGCGGTGGAGCATGTGGTTTAATTCGACGCAACGCGAGGAACCTTACCAGCGTTTGACATCCT-ACGAACGGTGCAG      7 588215|EU703281.1         72.4%      ACAAGCGGTGGAGCATGTGGCTTAATTCGATGCAACGCGAAGAACCTTACCTGGGCTTGACATGTT-AGTCGTAGACTTG      8 471149|NZ_ACUZ01000110.1  69.2%      ACAAGCGGAGGAACATGTGGTTTAATTCGATGATACGCGAGGAACCTTACCCGGGCTTGAATTGCAGACGAACGATTCAG        consensus/100%                       ACAAGCGG.GGAryATGTGGyTTAATTCGAyG..A.GCGArGAACCTTACC.r..yTTGA..T.y......r.....y.G        consensus/90%                        ACAAGCGG.GGAryATGTGGyTTAATTCGAyG..A.GCGArGAACCTTACC.r..yTTGA..T.y......r.....y.G        consensus/80%                        ACAAGCGGTGGAGCATGTGGTTTAATTCGAyGCAACGCGArGAACCTTACC.r..CTTGACAT.C.....AA...y.y.G        consensus/70%                        ACAAGCGGTGGAGCATGTGGTTTAATTCGAyGCAACGCGAAGAACCTTACC.GG.CTTGACAT.Cy ..GAA...y.yAG                                         1041          :         .         .         .         .         1         .         . 1120 1 J01695.2                 100.0%      AGATGAGAATGTG-CCTTCGGGA---ACCGTGAGACAGGTGCTGCATGGCTGTCGTCAGCTCGTGTTGTGAAATGTTGGG      2 160267|DQ229066.1         87.7%      AGATGAGTTTGTG-CCTTCGGGA---ACTTAGAGACAGGTGCTGCATGGCTGTCGTCAGCTCGTGTTGTGAAATGTTGGG      3 101027|NC_005973.1        72.0%      AAAGAGAGGGGTG-CCCTCGGGA---ACGCGGACACAGGTGGTGCATGGCTGTCGTCAGCTCGTGCCGTAAGGTGTTGGG      4 268298|NC_010337.2        75.3%      AGATGCGAGGGTGCCCTTCGGGG--AGCGGAGAGACAGGTGGTGCATGGTTGTCGTCAGCTCGTGTCGTGAGATGTTGGG      5 592884|NR_029335.1        73.5%      AGATATGTTGGAGGTTATCAGGG---------AGACAGGTGGTGCATGGTTGTCGTCAGCTCGTGTCGTGAGATGTTGGG      6 584703|NR_028933.1        72.8%      AGATGCGCCGGTGCCCCTTCGGGGGAACGTAGTGACAGGTGGTGCATGGCTGTCGTCAGCTCGTGTCGTGAGATGTTGGG      7 588215|EU703281.1         72.4%      TGAAAGCTTGTCGTCACTTCGGG--TGGACTATCACAGGTGCTGCATGGCTGTCGTCAGCTCGTGTCGTGAGATGTTGGG      8 471149|NZ_ACUZ01000110.1  69.2%      AGATGATGAGG---CCCTTCGGG---GCGTCTGTGAAGGTGCTGCATGGTTGTCGTCAGCTCGTGCCGTGAGGTGTCGGC        consensus/100%                       .rA.r.........y..Ty.GGr...........r.AGGTG.TGCATGGyTGTCGTCAGCTCGTGyyGTrArrTGTyGG.        consensus/90%                        .rA.r.........y..Ty.GGr...........r.AGGTG.TGCATGGyTGTCGTCAGCTCGTGyyGTrArrTGTyGG.        consensus/80%                        AGA.r.....G.G.CyyTy.GGr  .r.......ACAGGTG.TGCATGGyTGTCGTCAGCTCGTGyyGTGArrTGTTGGG        consensus/70%                        AGATr.r..GGyG.CCyTy.GGr  .rC...rr.ACAGGTG.TGCATGGyTGTCGTCAGCTCGTGTCGTGAGATGTTGGG                                         1121          .         .         :         .         .         .         .         2 1200 1 J01695.2                 100.0%      TTAAGTCCCGCAACGAGCGCAACCCTTATCCTTTGTTGCCAGCGGTCCG-GCCGGGAACTCAAAGGAGACTGCCAGTGAT      2 160267|DQ229066.1         87.7%      TTAAGTCCCGCAACGAGCGCAACCCTTATCCTTTGTTGCCAGCGATTCG-GTCGGGAACTCAAAGGAGACTGCCAGTGAT      3 101027|NC_005973.1        72.0%      TTAAGTCTCGCAACGAGCGCAACCCTCGTGTTTAGTTGCCACT--ATGA-GTTTGGAACCCTGAACAGACCGCCGGTGTT      4 268298|NC_010337.2        75.3%      TTAAGTCCCGCAACGAGCGCAACCCTTATCCTCAGTTGCCAGCGAGAGA-GACGGGGACTCTGGGGAGACTGCCCGGGAC      5 592884|NR_029335.1        73.5%      TTAAGTCCCGCAACGAGCGCAACCCTTGTCTTTAGTTNCTAAC-ATTAA-GTTGAGGACTCTAGAGAGACTGCCGGTGAC      6 584703|NR_028933.1        72.8%      TTAAGTCCCGCAACGAGCGCAACCCCTATCGTATGTTACCAGC-CTTCA-GTTGGGGACTCATGCGATACTGCCTGCGAC      7 588215|EU703281.1         72.4%      TTAAGTCCCGTAACGAGCGCAACCCCTATGAACTGTTGCCAGCGGGTAATGCCGGGGACTCTGTTCAGACTGCCCAGATT      8 471149|NZ_ACUZ01000110.1  69.2%      TTAAGTGCCATAACGAGCGCAACCCCTTTCTTCAGTTGCCATCAGGTGATGCTGGGCACTCTGTAGATACTGCCACCGCA        consensus/100%                       TTAAGT.yCryAACGAGCGCAACCCyy.T.....GTT.CyA.y.....r.G.y.rG.ACyC.....A.ACyGCC...r..        consensus/90%                        TTAAGT.yCryAACGAGCGCAACCCyy.T.....GTT.CyA.y.....r.G.y.rG.ACyC.....A.ACyGCC...r..        consensus/80%                        TTAAGTCCCGyAACGAGCGCAACCCyTrT..Ty.GTTrCCA.C...y.r.GyyGGGrACTC.r...A.ACTGCC.r.G.y        consensus/70%                        TTAAGTCCCGCAACGAGCGCAACCCyTrTCyTy.GTTGCCArC.r.T.A GyyGGGrACTC.rrrGAGACTGCC.GyG.y                                         1201          .         .         .         .         :         .         .         . 1280 1 J01695.2                 100.0%      AAACTGGAGGAAGGTGGGGATGACGTCAAGTCATCATGGCCCTTACGACCAGGGCTACACACGTGCTACAATGGCGCATA      2 160267|DQ229066.1         87.7%      AAACTGGAGGAAGGTGGGGATGACGTCAAGTCATCATGGCCCTTACGAGTAGGGCTACACACGTGCTACAATGGTGCATA      3 101027|NC_005973.1        72.0%      AAGCCGGAGGAAGGAGAGGATGAGGCCAAGTCATCATGCCCCTTATGCCCTGGGCGACACACGTGCTACAATGGGCGGGA      4 268298|NC_010337.2        75.3%      GACCGGGAGGAAGGCGGGGATGACGTCAAATCATCATGCCCCTTATGTCTTGGGCTACACACGTGCTACAATGGGCGGTA      5 592884|NR_029335.1        73.5%      AAACCGGAGGAAGGTGGGGATGACGTCAAATCATCATGCCCCTTATGACCTGGGCTACACACGTACTACAATGGCGGATA      6 584703|NR_028933.1        72.8%      GAGCAGGAGGAAGGTGGGGATGACGTCAAGTCATCATGCCCCTTATACGCTGGGCTACACACGTGCTACAATGGGTAGTA      7 588215|EU703281.1         72.4%      AGCTGGGAGGAAGGAGGGGATGACGTCAAGTCAGTATGGCCCTTACGCCCAGGGCTGCACACGTGCTACAATGTCCAGTA      8 471149|NZ_ACUZ01000110.1  69.2%      AGGTGTGAGGAAGGTGGGGATGACGTCAAATCAGCACGGCCCTTACGTCCGGGGCTACACACGTGTTACAATGGCCGGTA        consensus/100%                       rr.y..GAGGAAGG.GrGGATGA.GyCAArTCA.yAyG.CCCTTAyr..y.GGGC.rCACACGTryTACAATG....r.A        consensus/90%                        rr.y..GAGGAAGG.GrGGATGA.GyCAArTCA.yAyG.CCCTTAyr..y.GGGC.rCACACGTryTACAATG....r.A        consensus/80%                        rr.y.GGAGGAAGG.GGGGATGACGTCAArTCA.CATG.CCCTTAyG..y.GGGCTACACACGTGCTACAATGG...rTA        consensus/70%                        AArC.GGAGGAAGGyGGGGATGACGTCAArTCATCATG.CCCTTAyG.CC.GGGCTACACACGTGCTACAATGG..rrTA                                         1281          .         3         .         .         .         .         :         . 1360 1 J01695.2                 100.0%      CAAAGAGAAGCGACCTCGCGAGAGCAAGCGGACCTCATAAAGTGCGTCGTAGTCCGGATTGGAGTCTGCAACTCGACTCC      2 160267|DQ229066.1         87.7%      CAGAGGGTGACGAAGCCGCGAGGTGGAGTGAATCTCAGAAAGTGCATCTAAGTCCGGATTGGAGTCTGCAACTCGACTCC      3 101027|NC_005973.1        72.0%      CAAAGGGTCGCGATCTCGCGAGGGTGAGCTAACTCCAAAAACCCGTCCTCAGTTCGGATTGCAGGCTGCAACTCGCCTGC      4 268298|NC_010337.2        75.3%      CAAACCGAGGCGAAGCCGCGAGGCGGAGCGAACCGGAGAAAGCCGCTCACAGTTCGGATTGCTCTCTGCAACTCGAGAGC      5 592884|NR_029335.1        73.5%      CAACGAGAAGCAAGACAGTGATGTGGAGCAAAACTCAGAAAGTCCGTCTCAGTTCGGATTGAAGTCTGCAACCCGACTTC      6 584703|NR_028933.1        72.8%      CAGAGAGCCGCAAACCCGCGAGGGGGAGCAAATCTCAGAAAACTATTCTTAGTTCGGATTGTACTCTGCAACTCGAGTAC      7 588215|EU703281.1         72.4%      CAATGTGAACCGAGACCGCGAGGTGGAGGAAATCT-ATAAAACTGGGCTCAGTTCGGATTGCAGGCTGCAACTCGCCTGC      8 471149|NZ_ACUZ01000110.1  69.2%      CAGAGAGTTGATTTTGTGCAAACACGATCTAATCC-TTAAATCCGGTCCCAGTTCGGACTGGGGTCTGCAACCCGACCCC        consensus/100%                       CAr...G..........GyrA....rA...rA.y....AAA.y....C..AGTyCGGAyTG....CTGCAACyCG....C        consensus/90%                        CAr...G..........GyrA....rA...rA.y....AAA.y....C..AGTyCGGAyTG....CTGCAACyCG....C        consensus/80%                        CAr.G.G..rCrA..yyGCGArr..GAGy.AAyCy.A.AAA.y...yC.yAGTyCGGATTG.r..CTGCAACyCG..y.C        consensus/70%                        CArAGrG..GCrA..yCGCGAGG..GAGCrAAyCy.A.AAAryy..TCyyAGTTCGGATTG.AGTCTGCAACTCGACT.C                                         1361          .         .         .         4         .         .         .         . 1440 1 J01695.2                 100.0%      ATGAAGTCGGAATCGCTAGTAATCGTGGATCAG-AATGCCACGGTGAATACGTTCCCGGGCCTTGTACACACCGCCCGTC      2 160267|DQ229066.1         87.7%      ATGAAGTCGGAATCGCTAGTAATCGCGAATCAG-AATGTCGCGGTGAATACGTTCCCGGGCCTTGTACACACCGCCCGTC      3 101027|NC_005973.1        72.0%      ATGAAGCAGGAATCGCTAGTAATCGCCGGTCAGCCATACGGCGGTGAATCCGTTCCCGGGCCTTGTACACACCGCCCGTC      4 268298|NC_010337.2        75.3%      ATGAAGGCGGAATCGCTAGTAATCGCGGGTCAG-CATACCGCGGTGAATACGTTCCCGGGCCTTGTACACACCGCCCGTC      5 592884|NR_029335.1        73.5%      ATGAAGCCGGAATCGCTAGTAATCGCGGATCAG-CATGCCGCGGTGAATACGTTCTCGGGCCTTGTACACACCGCCCGTC      6 584703|NR_028933.1        72.8%      ATGAAGTTGGAATCGCTAGTAATCGCAAATCAGCAATGTTGCGGTGAATACGTTCTCGGGTCTTGTACACACCGCCCGTC      7 588215|EU703281.1         72.4%      ATGAAGTTGGAATCGCTAGTAATGGTACATCAGCTACGGTACCGTGAATACGTTCCCGGGTCTTGTACACACCGCCCGTC      8 471149|NZ_ACUZ01000110.1  69.2%      ACGAAGCTGGATTCGCTAGTAATCGCGCATCAGCCATGGCGCGGTGAATACGTTCCCGGGCCTTGTACACACCGCCCGTC        consensus/100%                       AyGAAG..GGA.TCGCTAGTAAT.Gy..rTCAG..Ayr..rC.GTGAAT.CGTTCyCGGGyCTTGTACACACCGCCCGTC        consensus/90%                        AyGAAG..GGA.TCGCTAGTAAT.Gy..rTCAG..Ayr..rC.GTGAAT.CGTTCyCGGGyCTTGTACACACCGCCCGTC        consensus/80%                        ATGAAGyyGGAATCGCTAGTAATCGyr.rTCAG..ATr.yrCGGTGAATACGTTCyCGGGyCTTGTACACACCGCCCGTC        consensus/70%                        ATGAAGyyGGAATCGCTAGTAATCGCrrATCAG..ATGyyGCGGTGAATACGTTCCCGGGCCTTGTACACACCGCCCGTC                                         1441          :         .         .         .         .         5         .         . 1520 1 J01695.2                 100.0%      ACACCATGGGAGTGGGTTGCAAAAGAAGTAGGTAGCT-TAACCTTC----GGGAGGGCGCTTACCACTTTGTGATTCATG      2 160267|DQ229066.1         87.7%      ACACCATGGGAGTGGGTTGTACCAGAAGTAGATAGCT-------------------------------------------      3 101027|NC_005973.1        72.0%      ACACTATAGGAGCTGGCCATGTTTGAAGTCATTACCCTTAACCGTA----AGGAGGGGGATGCCTAAGGCTAGGCTTGCG      4 268298|NC_010337.2        75.3%      ACACCACGAAAGTCGGCAACACCCGAAGTCGGTGCGC-TAACCGCA----AGGAGGCAGCCGCCGAAGGTGGGGTCGATG      5 592884|NR_029335.1        73.5%      AAACCATGAGAGTTGGCAATACCCGAAGCCGGTGGCC-TAACCTCGCAAGAGGAGGGAGCCGTCGAAGGTAGGGCTGATG      6 584703|NR_028933.1        72.8%      ACACCACGAGAGTGGGTTGCACCTGAAGTAGCAGGCC-TAACCTTA----GGGAAGGATGCTCCGAGGGTGTGGTTCGCG      7 588215|EU703281.1         72.4%      ACATCATGGAAGCCGCTCGCACCCGAAG----------TGTCTCCA----------------------------------      8 471149|NZ_ACUZ01000110.1  69.2%      AAGCCATGAAAGCCGGGGGTGCCTGAAGTCTGTGACC--------------GCAAGGAACGGCCTAGGGCAAAACCGGTG        consensus/100%                       A.ryyAyrrrAGy.G...ryr...GAAG....................................................        consensus/90%                        A.ryyAyrrrAGy.G...ryr...GAAG....................................................        consensus/80%                        A.ACCAyGrrAGy.GGy.ryryy.GAAGy....r..y ........    ..............................        consensus/70%                        ACACCATGrrAGy.GGy.ryACCyGAAGT.r.Tr.Cy Tr.Cy.y.    .G.ArG.......C.A...y..rryy.ryG                                         1521          .         .         :         .         .         . ] 1582 1 J01695.2                 100.0%      ACTGGGGTGAAGTCGTAACAAGGTAACCGTAGGGGAACCTGCGGTTGGATCACCTCCTTA--      2 160267|DQ229066.1         87.7%      --------------------------------------------------------------      3 101027|NC_005973.1        72.0%      ACTGGAGTGAAGTCGTAACAAGGTAGCCGTACTGGAAGGTGCGGCTGGATCACCTCCTTTT-      4 268298|NC_010337.2        75.3%      ATTGGGGTGAAGTCGTAACAAGGTAGCCGTATCGGAAGGTGCGGCTGGATCACCTCCTTTCT      5 592884|NR_029335.1        73.5%      ATTGGGGT------------------------------------------------------      6 584703|NR_028933.1        72.8%      ATTGGGGTGAAGTCGTAACAAGGTAGCCGTA-------------------------------      7 588215|EU703281.1         72.4%      --------------------------------------------------------------      8 471149|NZ_ACUZ01000110.1  69.2%      ATTGGGGCTAAGTCGTAACAAGGTAGCCGTACCGGAAGGTGCGGCTGGAACACCTCCTT---        consensus/100%                       ..............................................................        consensus/90%                        ..............................................................        consensus/80%                        .............................................................         consensus/70%                        AyTGGrGy.................................................... ``` |
