# Supplementary material for: Critical Issues in Mycobiota Analysis
Source: Front Microbiol. 2017 Feb 14;8:180. doi: 10.3389/fmicb.2017.00180 (PMC5306204; doi:10.3389/fmicb.2017.00180)
Supplement: Supplementary file 7 [file DataSheet2.ZIP › supplementary_data_sheet_S2B.html]

```
Reference sequence (1): gi|698379407|gb|KM576414.1|_Hydnum-sp.-LM1961
Identities normalised by aligned length.
Colored by: identity + property
```

|  |
| --- |
| ```                                                                             1 [        .         .         .         .         :         .         .         . 80  1 gi|698379407|gb|KM576414.1|_Hydnum-sp.-LM1961                    100.0%     ----------------------------------------------CATTATTGAATATTACTGGGGGCTGGATGCTGGC     2 gi|750678793|dbj|AB906685.1|_Hydnum-repandum-var.-repandum        75.6%     ----------------------CCGTAGGTGAACCTGCGGAAGGATCATTAATGGATT---ACAGGGGGTTGATGCTGGC     3 gi|750678788|dbj|AB906680.1|_Hydnum-albomagnum                    75.8%     ----------------------CCGTAGGTGAACCTGCGGAAGGATCATTAATGGGTTTACAGGGGGGGTTGATGCTGGC     4 gi|530330116|gb|KC293545.1|_Hydnum-magnorufescens                 73.3%     AGTAAAAGTCGTAACAAGGTTTCCGTAGGTGAACCTGCGGAAGGATCATTAATGGATTTTTACAGAGGGTTGATGCTGGC     5 gi|530330115|gb|KC293544.1|_Hydnum-ovoideisporum-f.-depauperatum  71.7%     AGTAAAAGTCGTAACAAGGTTTCAGTAGGTGAACCTGCGGAAGGATCATTAATGGATTTACAGAGGGGTTTGATGCTGGC       consensus/100%                                                              ..............................................CATTA.TGrrT......rGrGG.T.GATGCTGGC       consensus/90%                                                               ..............................................CATTA.TGrrT......rGrGG.T.GATGCTGGC       consensus/80%                                                               ......................C.GTAGGTGAACCTGCGGAAGGATCATTAATGGATTT..A.rGGGG.TTGATGCTGGC       consensus/70%                                                               ......................C.GTAGGTGAACCTGCGGAAGGATCATTAATGGATTT..A.rGGGG.TTGATGCTGGC                                                                                 81          .         1         .         .         .         .         :         . 160 1 gi|698379407|gb|KM576414.1|_Hydnum-sp.-LM1961                    100.0%     AGTCTATGATTGCATGTGCTCGCTCTCTTTAATCTACTTACACATGTGCACCTCA--ATTTTGAAGAC-----GAGGTAA     2 gi|750678793|dbj|AB906685.1|_Hydnum-repandum-var.-repandum        75.6%     AG--TTTAGCTGCATGTGCTCACTCT-TCTGGTTTATTTACACCTGTGCACTTAATTCTTTCAAAGGC-----AGGGTAG     3 gi|750678788|dbj|AB906680.1|_Hydnum-albomagnum                    75.8%     AG--TTTAACTGCATGTGCTCGCTCT-TCTGATTTATTTACACCTGTGCACTTGATTTTTTCAAAGGG-----AGGGTCG     4 gi|530330116|gb|KC293545.1|_Hydnum-magnorufescens                 73.3%     AG--TTTATCTGCATGTGCTCGCTCT-TCTGATTTATTTACACCTGTGCACTTAATTCTTTTCAAAGGCCTTAGGGGTAG     5 gi|530330115|gb|KC293544.1|_Hydnum-ovoideisporum-f.-depauperatum  71.7%     AGTTTTTATCTGTATGTGCTCACTCT-TCTGATTTATTTACACTTGTGCACTTAGTTTTTTCAAGGGTC----AGGATAG       consensus/100%                                                              AG..T.Tr.yTGyATGTGCTCrCTCT.TyTrrTyTAyTTACAC.TGTGCACyT.r...TTTy.Arrr......rrGrT.r       consensus/90%                                                               AG..T.Tr.yTGyATGTGCTCrCTCT.TyTrrTyTAyTTACAC.TGTGCACyT.r...TTTy.Arrr......rrGrT.r       consensus/80%                                                               AG..TTTA.CTGCATGTGCTCrCTCT TCTGATTTATTTACACyTGTGCACTTrATTyTTTyrAAGG..    rGGGTAG       consensus/70%                                                               AG..TTTA.CTGCATGTGCTCrCTCT TCTGATTTATTTACACyTGTGCACTTrATTyTTTyrAAGG..    rGGGTAG                                                                                161          .         .         .         2         .         .         .         . 240 1 gi|698379407|gb|KM576414.1|_Hydnum-sp.-LM1961                    100.0%     -AGCTTGTCTTTGGGATTTTATA--AACTCTTACT--CGATGTAATGAATGTTTTTGTCTGCCGAAAGGCAAAATTT---     2 gi|750678793|dbj|AB906685.1|_Hydnum-repandum-var.-repandum        75.6%     -AACCTGCCTTTGGAACT-TGTA--AACCCCTTGTATTGAAGCAATGAATG--TTTATCTGCCACAAGGCGAATTTT---     3 gi|750678788|dbj|AB906680.1|_Hydnum-albomagnum                    75.8%     CTCCCTGCCTTTGAAACT-TATA--AACCCCTTGTACTGAAATAATGAATG--TTTATCTGCCATGGGGCAAATTTT---     4 gi|530330116|gb|KC293545.1|_Hydnum-magnorufescens                 73.3%     AAACCTGCCTTTGGAGCTTTATA--AACCCCCTAT-TTGAGATTATGGATG--TTTATCTGCCACAAGGCAAAATTTT--     5 gi|530330115|gb|KC293544.1|_Hydnum-ovoideisporum-f.-depauperatum  71.7%     -AACCTGCCTTTGGAACT-TATAACACCCCCCTATATTGAAATAATGGATG--TTTATGTGCCGCAAGGCTTTATTTTTA       consensus/100%                                                              ...CyTGyCTTTGrrryT.TrTA..A.CyCyy..T..yGA.ry.ATGrATG..TTTrT.TGCCr.rrGGC....TTT...       consensus/90%                                                               ...CyTGyCTTTGrrryT.TrTA..A.CyCyy..T..yGA.ry.ATGrATG..TTTrT.TGCCr.rrGGC....TTT...       consensus/80%                                                               .ArCCTGCCTTTGGAACT.TATA  AACCCCyTrT.yTGArrTAATGrATG  TTTATCTGCCryAAGGCrAA.TTT.         consensus/70%                                                               .ArCCTGCCTTTGGAACT.TATA  AACCCCyTrT.yTGArrTAATGrATG  TTTATCTGCCryAAGGCrAA.TTT.                                                                                  241          :         .         .         .         .         3         .         . 320 1 gi|698379407|gb|KM576414.1|_Hydnum-sp.-LM1961                    100.0%     AATACAACTTTTAACAACGGATCTCTTGGCTCTCGCATCGATGAAGAACGCAGCGAAATGCGATAAGTAATGTGAATTGC     2 gi|750678793|dbj|AB906685.1|_Hydnum-repandum-var.-repandum        75.6%     AATACAACTTTTAACAACGGATCTCTTGGCTCTCGCATCGATGAAGAACGCAGCGAAATGCGATAAGTAATGTGAATTGC     3 gi|750678788|dbj|AB906680.1|_Hydnum-albomagnum                    75.8%     AATACAACTTTTAACAATGGATCTCTTGGCTCTCGCATCGATGAAGAACGCAGCGAAATGCGATAAGTAATGTGAATTGC     4 gi|530330116|gb|KC293545.1|_Hydnum-magnorufescens                 73.3%     AATACAACTTTTAACAACGGATCTCTTGGCTCTCGCATCGATGAAGAACGCAGCGAAATGCGATAAGTAATGTGAATTGC     5 gi|530330115|gb|KC293544.1|_Hydnum-ovoideisporum-f.-depauperatum  71.7%     TATACAACTTTTAACAACGGATCTCTTGGCTCTCGCATCGATGAAGAACGCAGCGAAATGCGATAAGTAATGTGAATTGC       consensus/100%                                                              .ATACAACTTTTAACAAyGGATCTCTTGGCTCTCGCATCGATGAAGAACGCAGCGAAATGCGATAAGTAATGTGAATTGC       consensus/90%                                                               .ATACAACTTTTAACAAyGGATCTCTTGGCTCTCGCATCGATGAAGAACGCAGCGAAATGCGATAAGTAATGTGAATTGC       consensus/80%                                                               AATACAACTTTTAACAACGGATCTCTTGGCTCTCGCATCGATGAAGAACGCAGCGAAATGCGATAAGTAATGTGAATTGC       consensus/70%                                                               AATACAACTTTTAACAACGGATCTCTTGGCTCTCGCATCGATGAAGAACGCAGCGAAATGCGATAAGTAATGTGAATTGC                                                                                321          .         .         :         .         .         .         .         4 400 1 gi|698379407|gb|KM576414.1|_Hydnum-sp.-LM1961                    100.0%     AGAATTCAGTGAATCATCGAATCTTTGAACGCACCTTGCGCTCTCTGGTATTCCGGAGAGTACGCCTGTTCGAGTGTCA-     2 gi|750678793|dbj|AB906685.1|_Hydnum-repandum-var.-repandum        75.6%     AGAATTCAGTGAATCATCGAATCTTTGAACGCACCTTGCGCTCTCTGGTATTCCGGGGAGCACACCTGTTCGAGTGTCAT     3 gi|750678788|dbj|AB906680.1|_Hydnum-albomagnum                    75.8%     AGAATTCAGTGAATCATCGAATCTTTGAACGCACCTTGCGCTCTCTGGTATTCCGGGGAGCACACCTGTTCGAGTGTCAT     4 gi|530330116|gb|KC293545.1|_Hydnum-magnorufescens                 73.3%     AGAATTCAGTGAATCATCGAATCTTTGAACGCACCTTGCGCTCTCTGGTATTCCGGGGAGCACACCTGTTCGAGTGTCAT     5 gi|530330115|gb|KC293544.1|_Hydnum-ovoideisporum-f.-depauperatum  71.7%     AGAATTCAGTGAATCATCGAATCTTTGAACGCACCTTGCGCTCTCTGGTATTCCGGGGAGCACGCCTGTTCGAGTGTCAT       consensus/100%                                                              AGAATTCAGTGAATCATCGAATCTTTGAACGCACCTTGCGCTCTCTGGTATTCCGGrGAGyACrCCTGTTCGAGTGTCA.       consensus/90%                                                               AGAATTCAGTGAATCATCGAATCTTTGAACGCACCTTGCGCTCTCTGGTATTCCGGrGAGyACrCCTGTTCGAGTGTCA.       consensus/80%                                                               AGAATTCAGTGAATCATCGAATCTTTGAACGCACCTTGCGCTCTCTGGTATTCCGGGGAGCACrCCTGTTCGAGTGTCAT       consensus/70%                                                               AGAATTCAGTGAATCATCGAATCTTTGAACGCACCTTGCGCTCTCTGGTATTCCGGGGAGCACrCCTGTTCGAGTGTCAT                                                                                401          .         .         .         .         :         .         .         . 480 1 gi|698379407|gb|KM576414.1|_Hydnum-sp.-LM1961                    100.0%     TGAAACTCTCAGGC-AGAGATAGCTTT--GTTGCTGTTTTTGTTTGGATTTGGACTTTGCTGTG---CCAATGCGGCTGG     2 gi|750678793|dbj|AB906685.1|_Hydnum-repandum-var.-repandum        75.6%     TGAAACTCTCAAAT-AAAGGTGGTTTTTGCAAACCATCTCTGTTTGGATTTGGACTTTGCTGCA---TTAATGTGGCTAG     3 gi|750678788|dbj|AB906680.1|_Hydnum-albomagnum                    75.8%     TGATACTCTCAAAC-AAAGGGAGCTTT--GTAGCTCTCTCTGTTTGGAATTGGACTCTGCTGCAATTATAATGTGGCTAG     4 gi|530330116|gb|KC293545.1|_Hydnum-magnorufescens                 73.3%     TAAAATCCTCAAAT-AAAGGTGGCTTT--GCAGCTATCTCTGTTTGGATTTGGGCTTTGCTGCATATTAATTGTGGCTAG     5 gi|530330115|gb|KC293544.1|_Hydnum-ovoideisporum-f.-depauperatum  71.7%     TAAAACTCTCAAATAAAAGGTGGCTTT--GCAGCTATCTCTGTTTGGATTTGGGCTTTGCTGCA-TTTTAGTGTGGCTAG       consensus/100%                                                              TrA.AyyCTCArry.ArAGr.rGyTTT.....rCy.TyTyTGTTTGGA.TTGGrCTyTGCTGyr.....A.TGyGGCTrG       consensus/90%                                                               TrA.AyyCTCArry.ArAGr.rGyTTT.....rCy.TyTyTGTTTGGA.TTGGrCTyTGCTGyr.....A.TGyGGCTrG       consensus/80%                                                               TrAAACTCTCAAAy AAAGGTrGCTTT  GyAGCTrTCTCTGTTTGGATTTGGrCTTTGCTGCA...yyArTGTGGCTAG       consensus/70%                                                               TrAAACTCTCAAAy AAAGGTrGCTTT  GyAGCTrTCTCTGTTTGGATTTGGrCTTTGCTGCA...yyArTGTGGCTAG                                                                                481          .         5         .         .         .         .         :         . 560 1 gi|698379407|gb|KM576414.1|_Hydnum-sp.-LM1961                    100.0%     TCTTAAATGTATTAGCTGGTCCTAATATGGGGGTTTTGGTTCTACTCAGCGTGATAATTATCTGACGCTGAGGACAGTCT     2 gi|750678793|dbj|AB906685.1|_Hydnum-repandum-var.-repandum        75.6%     TCTTAAATGTATTAGCAGATCCT--CATTGAGGTTTTGGTTCTACTCAGCATGATAATTATCTAATGTTGAGGACAGTTG     3 gi|750678788|dbj|AB906680.1|_Hydnum-albomagnum                    75.8%     TCTTAAATGTATTAGCTGGTCCT--CATTGAGGT-TTGGTTCTACTCAGCGTGATAATTATCTAACGTTGAGGACGGTCC     4 gi|530330116|gb|KC293545.1|_Hydnum-magnorufescens                 73.3%     TCTTAAATATATTAGCTGATCCT--TATTGAGGTTTTGGTTCTACTCAGCGTGGTAATTATCTAACGTTGAGGACAGTCT     5 gi|530330115|gb|KC293544.1|_Hydnum-ovoideisporum-f.-depauperatum  71.7%     TCTTAAATGTATTAGCTGATCCT--CATTGAGGTCTTGGTTCTACTCAGCGTGATAATTATCTAACGTTGAGGACAGTCT       consensus/100%                                                              TCTTAAATrTATTAGC.GrTCCT..yAT.GrGGT.TTGGTTCTACTCAGCrTGrTAATTATCTrAyGyTGAGGACrGTy.       consensus/90%                                                               TCTTAAATrTATTAGC.GrTCCT..yAT.GrGGT.TTGGTTCTACTCAGCrTGrTAATTATCTrAyGyTGAGGACrGTy.       consensus/80%                                                               TCTTAAATGTATTAGCTGrTCCT  yATTGAGGTyTTGGTTCTACTCAGCGTGATAATTATCTAACGTTGAGGACAGTCy       consensus/70%                                                               TCTTAAATGTATTAGCTGrTCCT  yATTGAGGTyTTGGTTCTACTCAGCGTGATAATTATCTAACGTTGAGGACAGTCy                                                                                561          .         .         .         6         .         .         .         . 640 1 gi|698379407|gb|KM576414.1|_Hydnum-sp.-LM1961                    100.0%     TAGGACTGGCCAGAGCTTATGTTTGGATTGCTTC--TAATTGTCCCTTGGACATACTGTTCAATTGTCTGACCTCCGGAT     2 gi|750678793|dbj|AB906685.1|_Hydnum-repandum-var.-repandum        75.6%     CAAGACTGGCCATGACTC-TCTTTGGATTGCTTC--TAAATCGTCTTAAGGACAATTGTTTAATTTTCTGACCT-CGAAT     3 gi|750678788|dbj|AB906680.1|_Hydnum-albomagnum                    75.8%     CAGGACTGGCCATGGCTC-TCTCTGGATTGCTTCT-TAAATGGTCTTGAGGACAATCACTCAA-TTTCTGACCT-CGAAT     4 gi|530330116|gb|KC293545.1|_Hydnum-magnorufescens                 73.3%     CAGAACTGGCCATGGCTC-TCTCTGGATTGCTTCT-AGACTGTCTTTAGGGACAATTGCTTAA-TTTCTGACCT-CGAAT     5 gi|530330115|gb|KC293544.1|_Hydnum-ovoideisporum-f.-depauperatum  71.7%     TAGAACTGGCCATAGCTC-TCTCTGGATTGCTTCTAAAAATTGTCTTGGGGACAAT------------------------       consensus/100%                                                              yArrACTGGCCA.rrCTy.T.TyTGGATTGCTTC...rA.T..yyyT.rGr...Ay........................       consensus/90%                                                               yArrACTGGCCA.rrCTy.T.TyTGGATTGCTTC...rA.T..yyyT.rGr...Ay........................       consensus/80%                                                               yAGrACTGGCCATrGCTC TCTyTGGATTGCTTC. .AA.T..yCTTrrGGACAATyryTyAA.T.TCTGACCT CGrAT       consensus/70%                                                               yAGrACTGGCCATrGCTC TCTyTGGATTGCTTC. .AA.T..yCTTrrGGACAATyryTyAA.T.TCTGACCT CGrAT                                                                                641          :         .         .  ] 673 1 gi|698379407|gb|KM576414.1|_Hydnum-sp.-LM1961                    100.0%     CAGGTGGGATTACCCGCTGAACTTAA-------     2 gi|750678793|dbj|AB906685.1|_Hydnum-repandum-var.-repandum        75.6%     CAGGTGGGACTACCCGCTGAACTTAAGCATATC     3 gi|750678788|dbj|AB906680.1|_Hydnum-albomagnum                    75.8%     CAGGTGGGACTACCCGCTGAACTTAAGCATATC     4 gi|530330116|gb|KC293545.1|_Hydnum-magnorufescens                 73.3%     CAGGTGGGACTACCCGCTGAACTTAAGC----T     5 gi|530330115|gb|KC293544.1|_Hydnum-ovoideisporum-f.-depauperatum  71.7%     ---------------------------------       consensus/100%                                                              .................................       consensus/90%                                                               .................................       consensus/80%                                                               CAGGTGGGAyTACCCGCTGAACTTAA.......       consensus/70%                                                               CAGGTGGGAyTACCCGCTGAACTTAA....... ``` |
